# TvLEGU-1 and TvLEGU-2 biomarkers for trichomoniasis are legumain-like cysteine peptidases secreted *in vitro* in a time-dependent manner

**DOI:** 10.3389/fpara.2025.1546468

**Published:** 2025-03-05

**Authors:** Esly Alejandra Euceda-Padilla, Miriam Guadalupe Mateo-Cruz, Jaime Ortega-López, Rossana Arroyo

**Affiliations:** ^1^ Departamento de Infectómica y Patogénesis Molecular, Centro de Investigación y de Estudios Avanzados del Instituto Politécnico Nacional (Cinvestav), Mexico City, Mexico; ^2^ Departamento de Biotecnología y Bioingeniería, Centro de Investigación y de Estudios Avanzados del Instituto Politécnico Nacional (Cinvestav), Mexico City, Mexico

**Keywords:** glucose, *in vitro* secretion, legumain-like cysteine peptidase, proteolytic activity, *Trichomonas vaginalis*, TvLEGU-1, TvLEGU-2

## Abstract

*Trichomonas vaginalis* is the causative agent of trichomoniasis, the most prevalent neglected parasitic sexually transmitted infection worldwide. Cysteine peptidases (CPs) are the most abundant proteins in the parasite degradome. Some CPs are virulence factors involved in trichomonal pathogenesis, cytoadherence, hemolysis, and cytotoxicity. Few are immunogenic and are found in the vaginal secretions of patients with trichomoniasis. Legumains are CPs of the C13 family of clan CD. *T. vaginalis* has 10 genes encoding legumain-like peptidases, and TvLEGU-1 and TvLEGU-2 have been characterized. Both are immunogenic and found in the vaginal secretions of patients with trichomoniasis that could be considered as potential biomarkers. Thus, our goal was to evaluate the effects of glucose on the proteolytic activity and secretion processes of TvLEGU-1 and TvLEGU-2. We performed *in vitro* secretion assays using different glucose concentrations, examined the presence and proteolytic activity of secreted legumains by Western blot and spectrofluorometry assays, and analyzed the localization of TvLEGU-1 and TvLEGU-2 in the parasites by indirect immunofluorescence. Our results show that TvLEGU-1 and TvLEGU-2 were secreted *in vitro* in a time-dependent manner and had legumain-like proteolytic activity that could contribute to parasite pathogenesis, supporting their relevance during infection and potential as trichomoniasis biomarkers.

## 1 Introduction

*Trichomonas vaginalis* is a unicellular parasite that causes trichomoniasis, a nonviral sexually transmitted disease with the highest incidence worldwide, with approximately ∼110 million new cases annually according to the WHO (Rowley et al., 2019). Complications associated with trichomoniasis include pelvic inflammatory disease, pyosalpinx, preterm delivery, posthysterectomy infection, infertility, and increased risk of cervical cancer (Petrin et al., 1998; Soper, 2004; Viikki et al., 2000; Zhang et al., 1995).

In the extensive degradome of *T. vaginalis* (~440 genes encoding peptidases), half encode cysteine peptidases (CPs), and some are virulence factors involved in the pathogenesis of trichomonads. These CPs are highly regulated by environmental factors such as pH, iron, glucose, zinc, and polyamines (Arroyo et al., 2015).

*T. vaginalis* has ten genes encoding legumain-like CPs (TvLEGU-1 to TvLEGU-10) (Carlton et al., 2007), of which only TvLEGU-1 and TvLEGU-2 have been characterized (Rendón-Gandarilla et al., 2013; Euceda-Padilla et al., 2024). Both peptidases are localized in the endolysosomal system, on the cell surface, secreted *in vitro* and *in vivo*, recognized by the immune system, and identified in the vaginal secretions of trichomoniasis patients (Rendón-Gandarilla et al., 2013; Miranda-Ozuna et al., 2019; Euceda-Padilla et al., 2024). Only TvLEGU-1 has been identified for its key role in *T. vaginalis* adherence to host epithelial cells (Rendón-Gandarilla et al., 2013).

Therefore, the main goal of this work was to study the *in vitro* secretion process of TvLEGU-1 and TvLEGU-2 under the influence of glucose. Our data revealed that mature TvLEGU-1 and TvLEGU-2 peptidases were secreted *in vitro* in a time-dependent manner through different parasite regions. Secreted peptidases retained their proteolytic activity that could contribute to parasite pathogenesis.

## 2 Materials and methods

### 2.1 Parasite culture

A fresh clinical CNCD 188 *T. vaginalis* isolate from one week of *in vitro* culture was used in this study (Alvarez-Sanchez et al., 2000). Parasites were cultured at 37°C in tryptone-yeast extract (TY) medium supplemented with 10% heat-inactivated adult bovine serum (HIBS) and glucose (D (+)-anhydrous glucose; Cat. No. 15866, Merck, Darmstadt, Germany) to a final concentration of 25 mM (normal glucose; NG). Trichomonads were also grown in 50 mM glucose (high glucose; HG) or without glucose (glucose restriction; GR), as previously reported (Miranda-Ozuna et al., 2016).

### 2.2 Secretion assays

The *in vitro* secretion assays were performed as described by Miranda-Ozuna et al. (2019). Briefly, parasites grown under HG or GR conditions were harvested, washed three times with PBS pH 7, and suspended (4 × 10^6^ parasites mL^−1^) in PBS pH 7 supplemented with 50 mM glucose (HG) or without glucose (GR). The parasites were incubated at 37°C for 0, 30, and 60 min. Parasite viability was measured using the trypan blue exclusion method. The supernatant containing the secretion products (SPs) was clarified by centrifugation at 900 × g. The SPs alone or after 10% TCA precipitation overnight (O/N) at 4°C were used for proteolytic activity assays or separated via SDS–PAGE on 10% polyacrylamide gels, blotted onto nitrocellulose (NC) membranes, and analyzed by Western blot (WB) assays. To obtain a protease-resistant extract (PRE) for enzymatic assays, parasites were lysed with 1% Triton X-100 (Thermo Scientific-Pierce, Rockford, IL, USA) and used as a control for total legumain proteolytic activity.

### 2.3 Legumain proteolytic activity

The legumain proteolytic activity of PRE or SPs was measured according to the protocol described by Reséndiz-Cardiel et al. (2017). The mixture was brought to 200 µL in citrate buffer (39.5 mM citric acid, 121 mM Na_2_HPO_4_ (pH 5.8), 1 mM DTT, 1 mM EDTA, 0.01% CHAPS), the fluorogenic substrate for legumains Z-Ala-Ala-Ala-Asn-MCA (benzyloxycarbonyl-L-alanyl-L-alanyl-L-alanyl-L-asparagine-4-methyl-coumaryl-7-amide) (Code: 3209-v, Peptide Institute Inc., Osaka, Japan), and 10 µg of PRE or 100 µL of SPs. The reaction was initiated by adding the fluorogenic substrate to a 10 µM final concentration, in a dark-bottomed 96-well microplate (Nunc) at 30°C. Immediately after substrate addition, the fluorescence released by substrate hydrolysis was measured at 360 nm excitation and 460 nm emission, using a Gemini EM spectrofluorimeter (Molecular Devices, Sunnyvale, CA, USA). As controls for legumain activity, E-64 (0.18 mM) and TLCK (1 mM) were used as inhibitors of papain and legumain-type peptidases, respectively (Coombs and North, 1983).

### 2.4 Western blot analysis

Total protein extracts (TPE) were obtained by 10% TCA precipitation. Proteins from TPE, SPs, or purified recombinant TvLEGU-1 (TvLEGU-1r) or TvLEGU-2 (TvLEGU-2r) (Ramón-Luing et al., 2010; Rendón-Gandarilla et al., 2013; Euceda-Padilla et al., 2024) were transferred onto 0.22 µm NC membranes (Bio-Rad, Hercules, CA, USA) for the WB analyses. The membranes were blocked with 5% nonfat milk for 18 h at 4°C, incubated with Rα-TvLEGU-1r (1:3000), or Rα-TvLEGU-2r (1:1000) antibody, or with secondary antibody alone, as a negative control for 18 h at 4°C, washed five times with PBS-0.1% Tween 20, incubated with a peroxidase-conjugated goat anti-rabbit secondary antibody (1:5000 dilution; Invitrogen, Waltham, MA, USA) for 1 h at 37°C, and developed with an enhanced chemiluminescence system (SuperSignal Chemiluminescent Substrate West Pico; Thermo Scientific-Pierce) in a Chemidoc XR (Bio-Rad).

The specificity of the Rα-TvLEGU-1r and Rα-TvLEGU-2r antibodies was evaluated by cross-recognition WB assays between TvLEGU-1 and TvLEGU-2 using NC membranes containing the recombinant proteins previously expressed in *E. coli*, TvLEGU-1r (Ramón-Luing et al., 2010) and TvLEGU-2r (Euceda-Padilla et al., 2024). The NC membranes were incubated with either Rα-TvLEGU-1r or Rα-TvLEGU-2r antibodies (**Supplementary Figure S1**). ImageLab software 5.0 (Bio-Rad) was used for all molecular weight analyses. Moreover, mouse and rabbit antibodies against trichomonad vesicles (α-TvVes) were generated and used as vesicle markers in indirect immunofluorescence assays (IFAs) and WB assays. The vesicles were obtained by ultracentrifugation at 100,000 x g for 60 min from parasite secretion products after 60 min under HG conditions. The resulting pellets were immunized with TiterMax Gold adjuvant twice, with a 15-day interval between immunizations. The specificity of the α-TvVes antibodies was assessed through WB assays using various proteins as antigens, including TvLEGU-1r, TvLEGU-2r, TPE, and SPs from parasites under both GR and HG conditions. Both antibodies gave similar recognition, and representative results are shown in **Supplementary Figure S2**.

### 2.5 Indirect immunofluorescence assays

*In vitro* secretion assays were performed as described above with some modifications. Parasites grown under HG and GR conditions and incubated at 37°C for 0, 30, and 60 min were processed for single- or double-step IFA, according to Sánchez-Rodríguez et al. (2018). Briefly, parasites were fixed with 4% formaldehyde and washed with 20 mM NH_4_Cl/PBS and 0.2% BSA in PBS (BSA/PBS). For single-step IFA, fixed parasites were permeabilized with 0.2% Triton X-100 for 10 min, washed with PBS and BSA/PBS, incubated with different primary antibodies, and washed and incubated with the corresponding secondary antibodies. For the double-step protocol, fixed parasites were directly incubated with the Rα-TvLEGU-1r or Rα-TvLEGU-2r primary antibody (1:300 or 1:100 dilution, respectively) for 2 h at room temperature (RT), washed with BSA/PBS and incubated with Mα-TvVes primary antibody (1:100 dilution) for 2 h at RT. Subsequently, the parasites were washed with BSA/PBS and incubated with FITC- and Alexa-Fluor 647-conjugated secondary antibodies (1:100 and 1:300 dilution, respectively) (Thermo-Scientific-Pierce) for 1 h at RT, washed with BSA/PBS and PBS, fixed again with 4% formaldehyde and washed with 20 mM NH_4_Cl/PBS and PBS. They were then permeabilized with 0.2% Triton X-100 for 10 min, washed with PBS and BSA/PBS, and incubated, as described above, with Rα-TvLEGU-1r, Rα-TvLEGU-2r and Mα-TvVes primary antibodies and the corresponding secondary antibodies or with secondary antibody alone as a negative control, using parasites at time 0 in all the IFA kinetics. Because at this time, we observed the highest fluorescent signal inside the parasites for both peptidases and the purpose of the secretion kinetic was to show the gradual loss of fluorescent signal for each protein. Nuclei were stained in all samples by mounting coverslips with Vectashield- 4′,6-diamidino-2-phenylindole (DAPI) mounting solution (Vector Laboratories, Burlingame, CA, USA). Parasites were analyzed by confocal microscopy using a Zeiss microscope and Zen Axio Imager Z2 software 3.8 (Carl Zeiss, Germany). Additionally, the fluorescence signal was quantified for each time and glucose condition in biological and technical duplicates to obtain the Pearson’s coefficient, evaluating between 34 and 97 parasites (please see **Supplementary Material**).

### 2.6 Statistical analysis

Statistically significant differences between means were determined by analysis of variance (Student’s t-test) using GraphPad Prism 8.0.1 and Sigma Plot 14.5. Scores with statistical significance are indicated by asterisks in the figures. The corresponding *P* values are shown in figure legends.

## 3 Results

### 3.1 The secretion products showed legumain-like proteolytic activity

**Figure 1:**
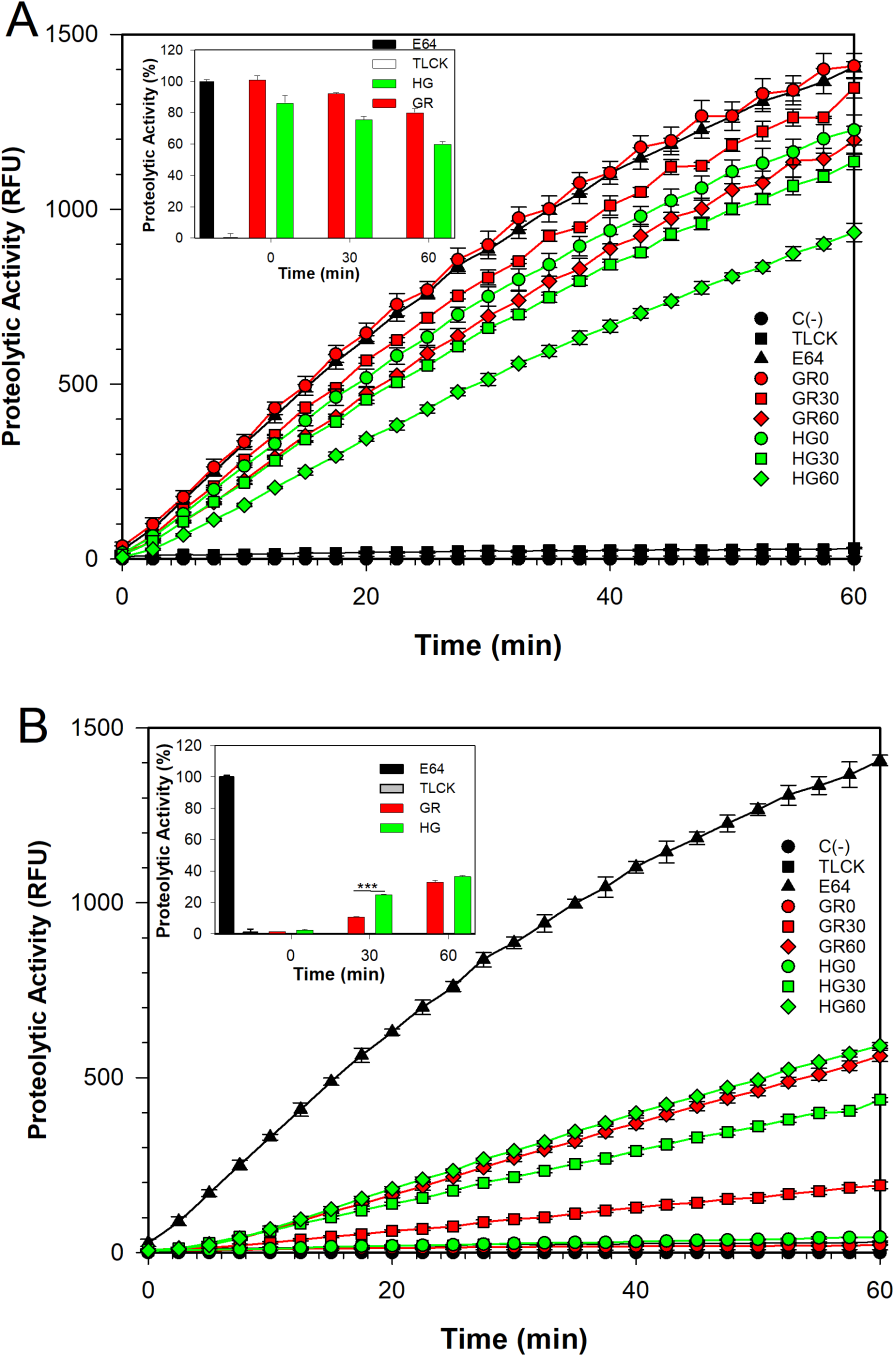
Legumain proteolytic activity at different glucose concentrations. Proteolytic activity profile of the protease-resistant extract (PRE) **(A)** and the secreted products (SPs) **(B)** of *T. vaginalis* grown under varying glucose conditions: glucose restriction (GR, indicated in red) and high glucose (HG, indicated in green). The activity was analyzed at three incubation times in secretion buffer: 0 (○), 30 (◻), and 60 (◇) min. Assays were performed as described in the Materials and Methods section. Legumain activity is reported in relative fluorescence units (RFCs). C (-) Assay buffer with the fluorogenic substrate Z-Ala-Ala-Ala-Asn-MCA (●), E-64 (▲), and TLCK (◼) were used as negative and positive controls of legumain proteolytic activity, respectively. The legumain enzyme activity was assessed by calculating the maximum slope of each activity profile using linear regression between 5 and 25 minutes. Insets show the percentage of legumain proteolytic activity, using PRE (GR) + E-64 enzyme activity as 100%. Statistical analysis was performed using ANOVA in SigmaPlot 14.5. (***) indicate statistically significant differences (P= <0.001).

To determine whether metabolically active *T. vaginalis* indeed secreted TvLEGU-1 and TvLEGU-2, we performed *in vitro* secretion kinetics to analyze legumain proteolytic activity, secreted isoforms, and localization during secretion by spectrofluorometry, WB, and IFAs, respectively. We first evaluated the proteolytic activity of the parasite extracts and SPs at 0, 30, and 60 min using the secretion assays to determine whether these secreted peptidases possessed legumain proteolytic activity. **Figure 1** shows that, over time from the onset of secretion to 60 min, the legumain activity was reduced in the parasite extracts (**Figure 1A**). These differences were statistically significant between 0 and 60 min of secretion, and a greater reduction in activity was observed in the HG (26.3%) than in the GR (20.8%) condition. In addition, we observed a gain of legumain activity in the SP under both glucose conditions (**Figure 1B**), which was statistically significant between 0 and 60 min of secretion, with greater activity at 30 min in the HG (22.5%) than in the GR (9.2%) condition. These results were consistent with the reduction in enzymatic activity of the parasite extracts. Over time, this increase in the legumain proteolytic activity of the parasite SPs suggested that secreted legumains could play an important role during *T. vaginalis* infection under different glucose conditions.

### 3.2 The secretion products contain both TvLEGU-1 and TvLEGU-2 peptidases

**Figure 2:**
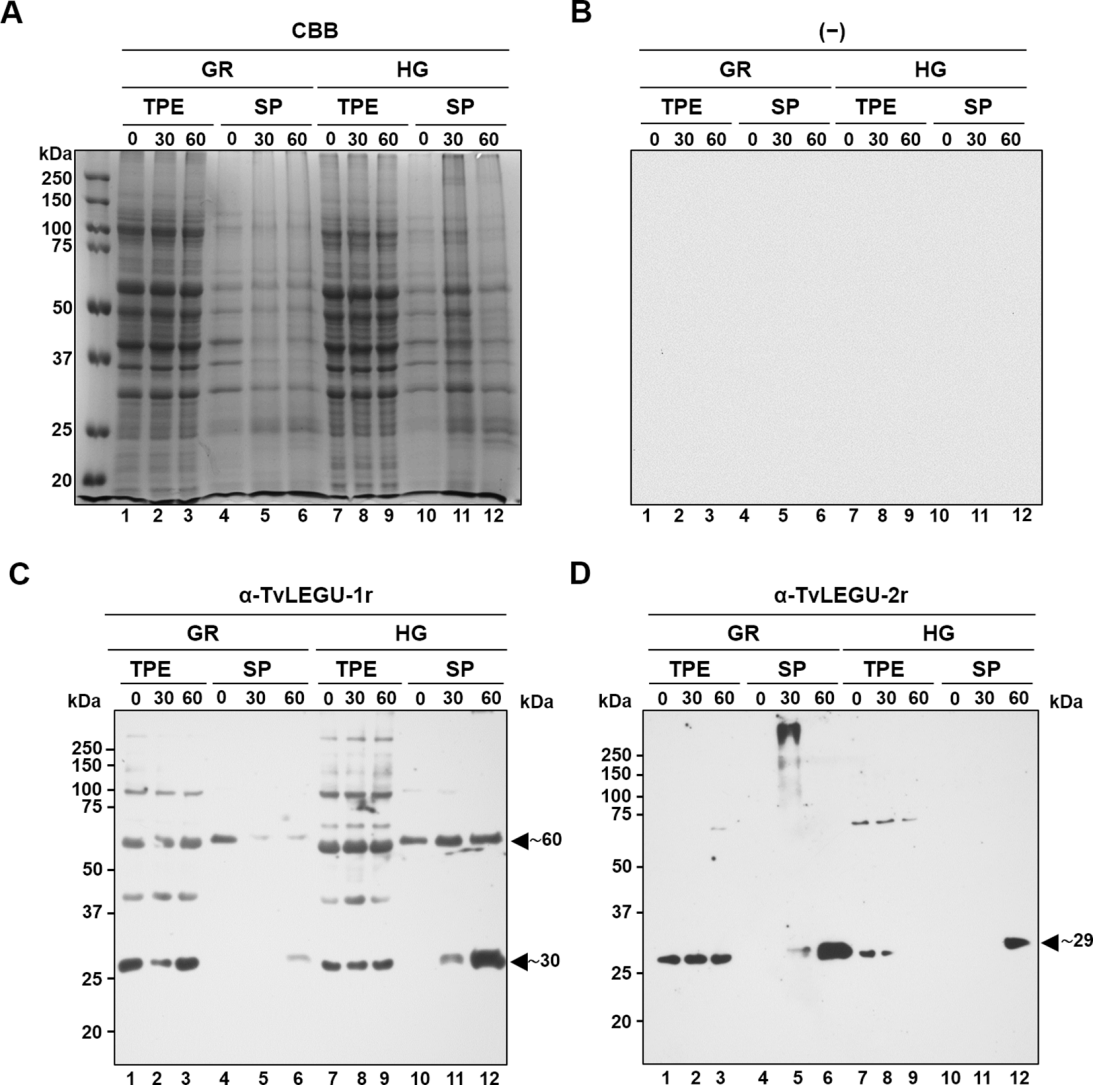
Glucose differentially regulates the *in vitro* secretion of mature TvLEGU-1 and TvLEGU-2 peptidases in *Trichomonas vaginalis*. **(A)** Kinetics of parasite secretion under GR and HG conditions at 0, 30, and 60 min analyzed by SDS–PAGE on Coomassie brilliant blue-stained (CBB) 10% polyacrylamide gels and by WB assays of proteins transferred onto NC membranes (lanes 1-12). **(B)** Negative control for WB was performed by adding only the secondary antibody to the NC membrane (-). **(C)** WB assay of total protein extracts (TPE) and secretion products (SPs) transferred onto NC membranes incubated with Rα-TvLEGU-1r antibody (1:3000 dilution) to detect the mature TvLEGU-1 protein (lanes 1-12) with an expected size of 30 kDa and possible other bands of the zymogen (expected size of 43 kDa) and processing steps with some PTMs (expected sizes 60 and 50 kDa). The arrowheads show the positions of the TvLEGU-1 proteins (∼30 and ∼60 kDa). **(D)** WB assay of TPE and SPs transferred onto NC membranes incubated with Rα-TvLEGU-2r antibody (1:1000 dilution) to detect the mature TvLEGU-2 protein (lanes 1-12) with an expected size of 29 kDa and possible other bands of the zymogen (expected size of 45 kDa) and processing steps with some PTMs (expected sizes of 55, 44, and 34 kDa). The arrowhead shows the position of the TvLEGU-2 protein (∼29 kDa). kDa, molecular weight marker in kilodaltons (Bio-Rad).

Since TvLEGU-1 and TvLEGU-2 are the most abundant legumains in the parasite (Dias-Lopes et al., 2018; Ramón-Luing et al., 2010) and are actively secreted, we performed WB assays of the parasite TPE and SPs at 0, 30, and 60 min, which were separated by SDS-PAGE in polyacrylamide CBB-stained gels (**Figure 2A**), or transferred onto NC membranes and incubated with specific anti-legumain antibodies, or with secondary antibodies alone, as a negative control (**Figure 2B**) to determine the amount and isoform of secreted TvLEGU-1 and TvLEGU-2 proteins (**Figures 2C, D**, respectively). For TvLEGU-1 under the GR condition, a significant peptidase loss was detected only after 30 min with recovery after 60 min of secretion of the mature protein (∼30 kDa) in the TPE, whereas the ∼30 and ∼60 kDa light bands of TvLEGU-1 were detected in the SPs at 60 min (**Figure 2C**, lanes 1-6). TvLEGU-2 under GR conditions did not show significant loss in the TPE, but a gain in SPs at 30 and 60 min of the 29 kDa band was observed (**Figure 2D**, lanes 1-6). In TvLEGU-1 under HG conditions bands of ∼30 kDa and ∼60 kDa were detected in TPEs (**Figure 2C**). In the SPs, the ∼60 kDa protein band at 0 min and ∼30 and ∼60 kDa bands at 30 and 60 min were observed (**Figure 2C**, lanes 7-12). For TvLEGU-2 under HG conditions, a gradual loss of protein in TPEs, mainly of the ∼29 kDa band, and a gain of the mature peptidase of 29 kDa at 60 min of secretion were observed (**Figure 2D**, lanes 7-12). Thus, both legumains were secreted under different glucose conditions and were found in the SPs. TvLEGU-1 was secreted mainly under HG conditions, whereas TvLEGU-2 was secreted mainly under GR conditions.

### 3.3 Antibodies against TvLEGU-1, TvLEGU-2, and trichomonad vesicles are specific

**Figure 3:**
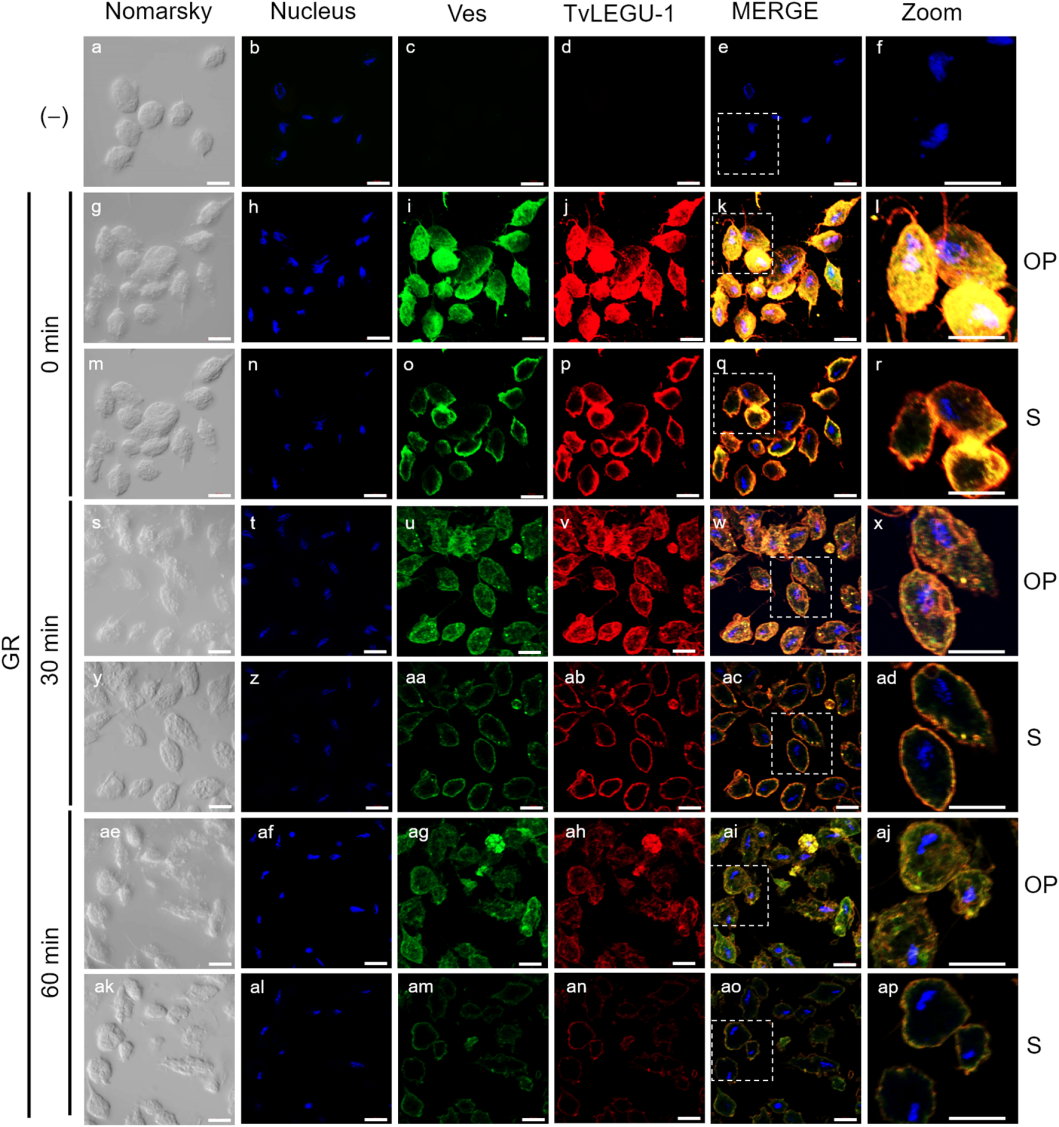
TvLEGU-1 is secreted in a time-dependent manner from parasites under glucose restriction (GR) conditions. Indirect immunofluorescence assays (IFAs) of permeabilized parasites under GR conditions **(g-ap)** with Rα-TvLEGU-1r (1:300 dilution) and Mα-TvVes (1:100 dilution) antibodies to label vesicles. Negative control was performed by adding only the secondary antibody (-) **(a-f)**. Orthogonal projection (OP): 0 min **(g-l)**, 30 min **(s-x)**, and 60 min **(ae-aj)**. Optical sections (S): 0 min **(m-r)**, 30 min **(y-ad)**, and 60 min **(ak-ap)**. Nuclei (DAPI, blue), vesicles (FITC, green), and TvLEGU-1 (Alexa 647, red) are shown. The white box in MERGE marks the magnified location (Zoom). White bar: 10 μm.

**Figure 4:**
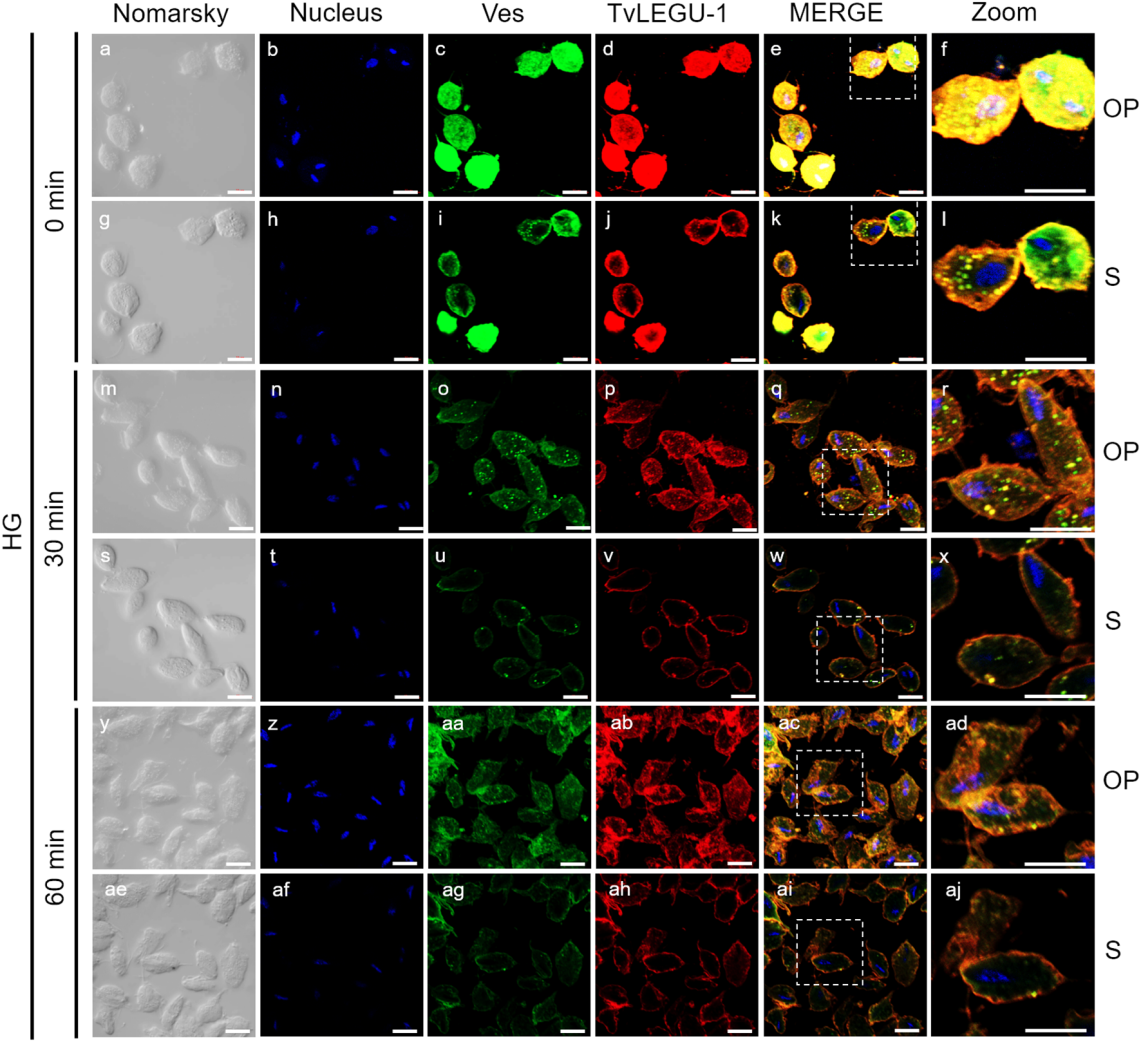
TvLEGU-1 is secreted in a time-dependent manner from parasites under high glucose (HG) conditions. IFA of parasites under HG conditions **(a-aj)** with Rα-TvLEGU-1r (1:300 dilution) and Mα-TvVes (1:100 dilution) antibodies to label vesicles. Orthogonal projection (OP): 0 min **(a-f)**, 30 min **(m-r)**, and 60 min (y-ad). Optical sections (S): 0 min **(g-l)**, 30 min **(s-x)**, and 60 min **(ae-aj)**. Nuclei (DAPI, blue), vesicles (FITC, green), and TvLEGU-1 (Alexa 647, red) are shown. The white box in MERGE marks the magnified location (Zoom). White bar, 10 μm.

**Figure 5:**
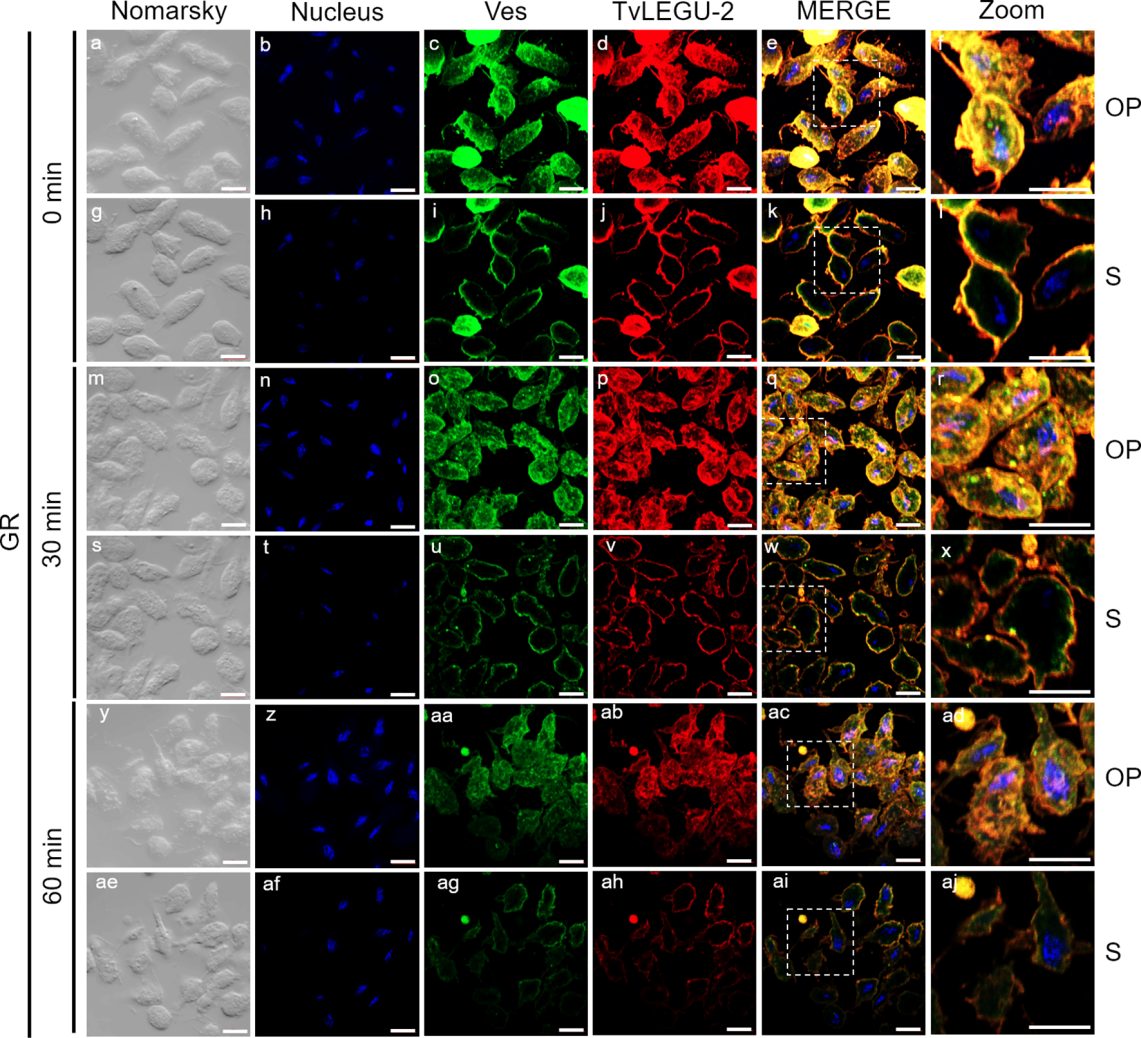
TvLEGU-2 is secreted in a time-dependent manner from parasites under glucose restriction (GR) conditions. IFA of permeabilized parasites under GR conditions **(a-aj)** with Rα-TvLEGU-2r (1:100 dilution) and Mα-TvVes (1:100 dilution) antibodies. Orthogonal projection (OP): 0 min **(a-f)**, 30 min **(m-r)**, and 60 min **(y-ad)**. Optical sections (S): 0 min **(g-l)**, 30 min **(s-x)**, and 60 min **(ae-aj)**. Nuclei (DAPI, blue), vesicles (FITC, green), and TvLEGU-2 (Alexa 647, red) are shown. The white box in MERGE marks the magnified location (Zoom). White bar: 10 μm.

**Figure 6:**
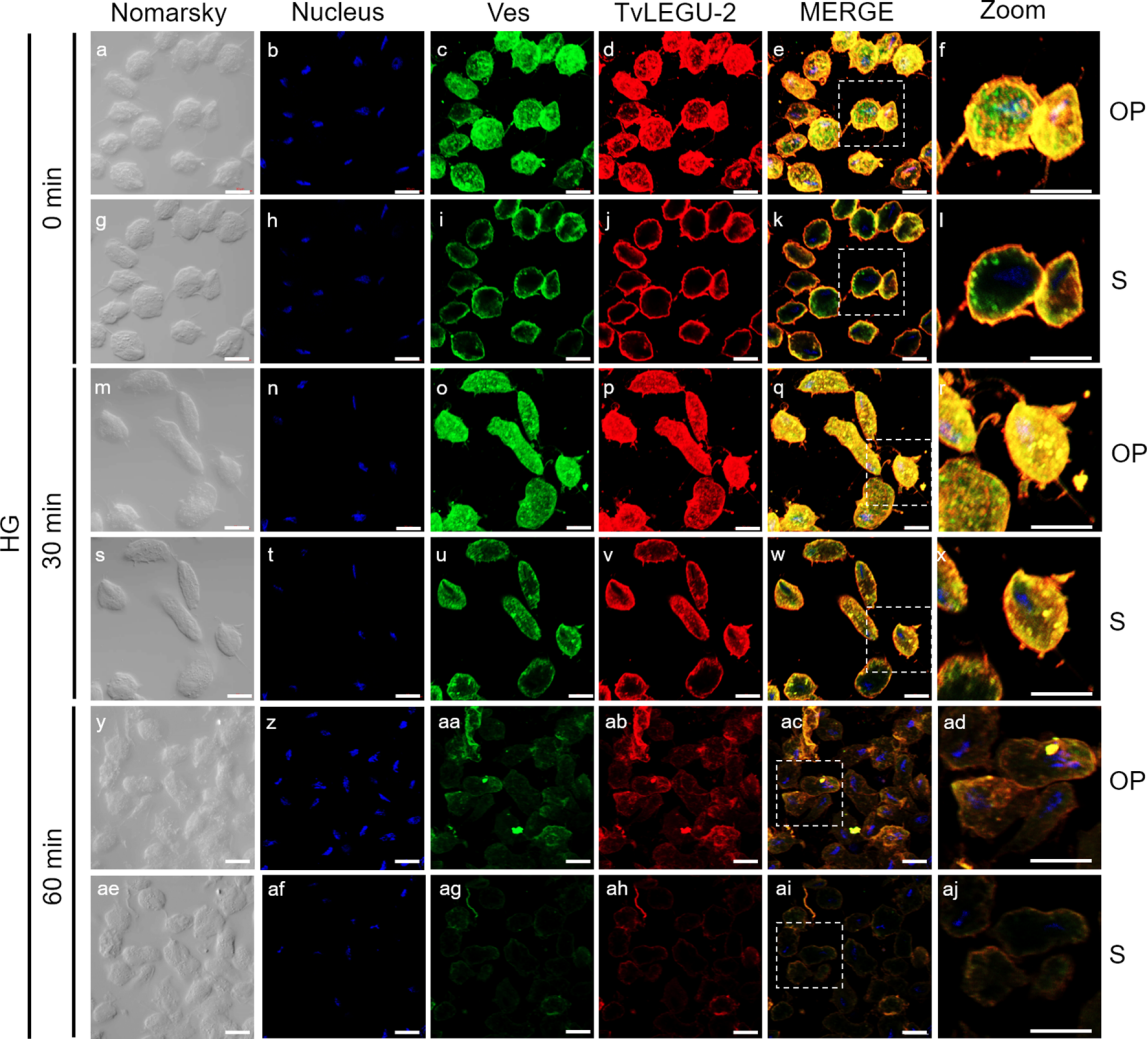
TvLEGU-2 is secreted in a time-dependent manner from parasites under high glucose (HG) conditions. IFA of parasites under HG conditions **(a-aj)** with Rα-TvLEGU-2r (1:100 dilution) and Mα-TvVes (1:100 dilution) antibodies. Orthogonal projection (OP): 0 min **(a-f)**, 30 min **(m-r)**, and 60 min **(y-ad)**. Optical sections (S): 0 min **(g-l)**, 30 min **(s-x)**, and 60 min **(ae-aj)**. Nuclei (DAPI, blue), vesicles (FITC, green), and TvLEGU-2 (Alexa 647, red) are shown. The white box in MERGE marks the magnified location (Zoom). White bar: 10 μm.

**Figure 7:**
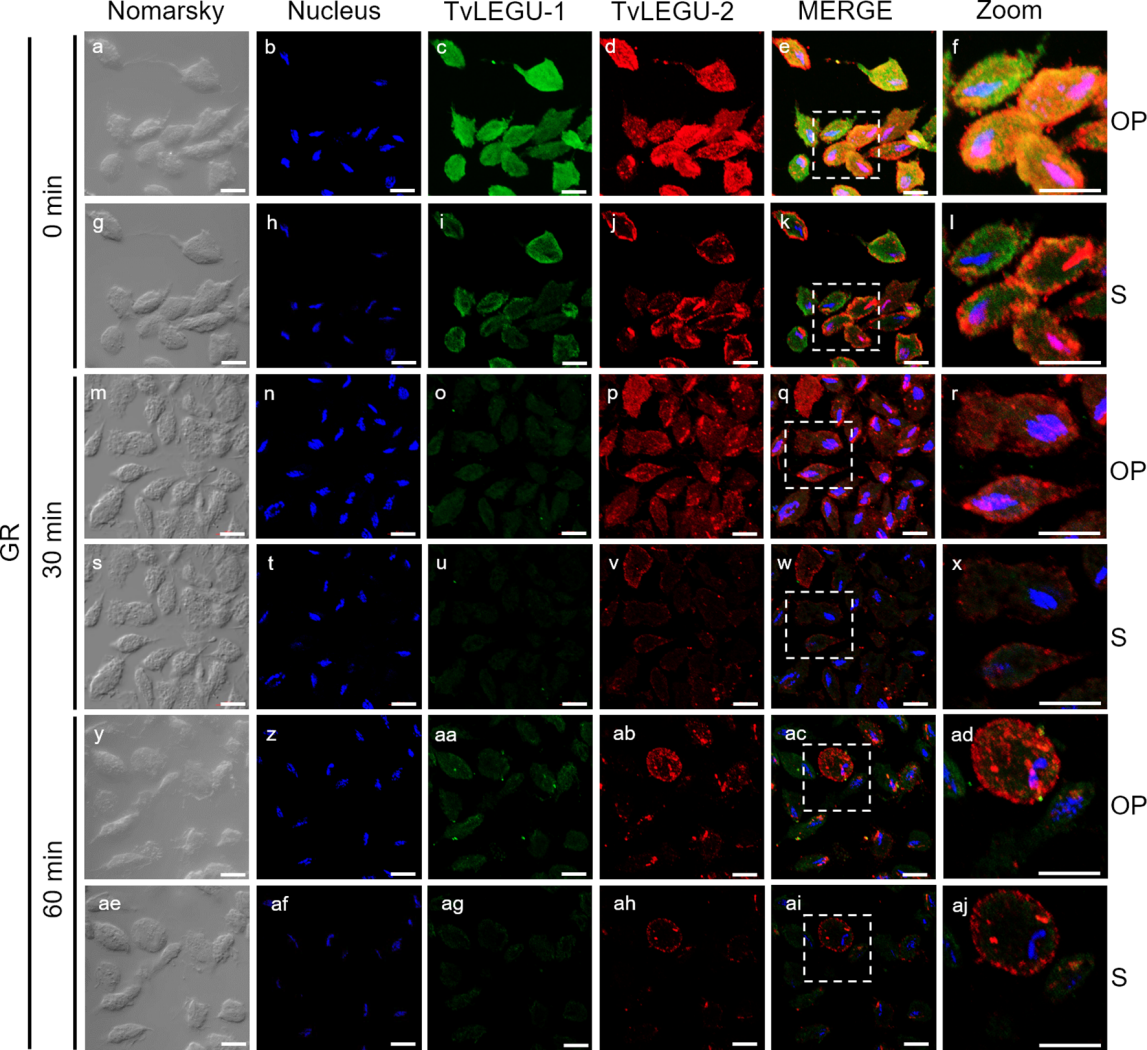
Colocalization of TvLEGU-1 and TvLEGU-2 during *T. vaginalis* secretion kinetics under glucose restriction (GR) conditions. IFA of permeabilized parasites under GR conditions **(a-aj)** with Rα-TvLEGU-1r (1:300 dilution) and Mα-TvLEGU-2r (1:100) antibodies. Orthogonal projection (OP): 0 min **(a-f)**, 30 min **(m-r)**, and 60 min (y-ad). Optical sections (S): 0 min **(g-l)**, 30 min **(s-x)**, and 60 min **(ae-aj)**. Nuclei (DAPI, blue), TvLEGU-1 (FITC, green), and TvLEGU-2 (Alexa 647, red) are shown. The white box in MERGE marks the magnified location (Zoom). White bar: 10 μm.

**Figure 8:**
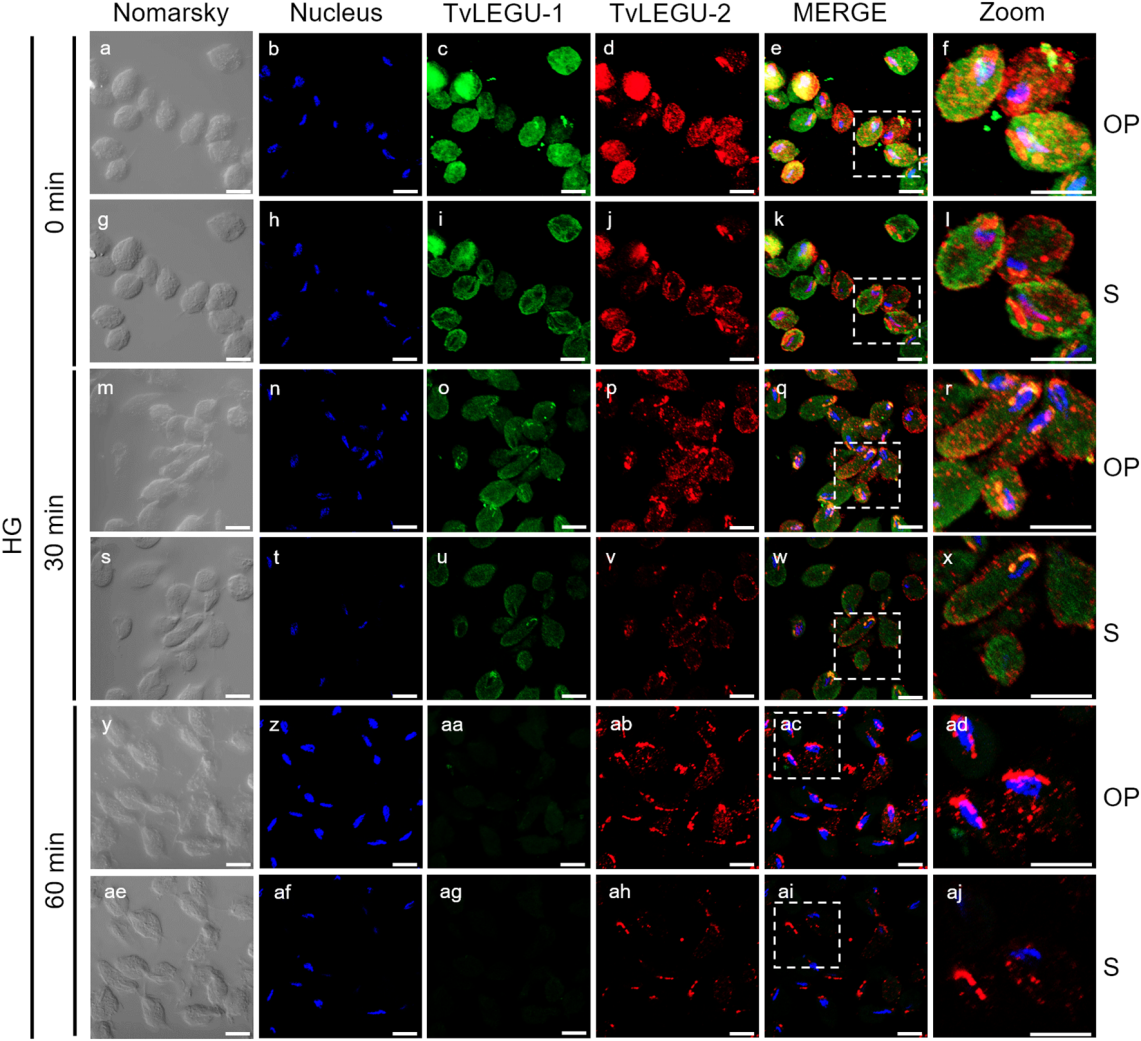
Colocalization of TvLEGU-1 and TvLEGU-2 during *T. vaginalis* secretion kinetics under high glucose (HG) conditions. IFA of parasites under HG conditions **(a-aj)** with Rα-TvLEGU-1r (1:300 dilution) and Mα-TvLEGU-2r (1:100) antibodies. Orthogonal projection (OP): 0 min **(a-f)**, 30 min **(m-r)**, and 60 min **(y-ad)**. Optical sections (S): 0 min **(g-l)**, 30 min **(s-x)**, and 60 min **(ae-aj)**. Nuclei (DAPI, blue), TvLEGU-1 (FITC, green), and TvLEGU-2 (Alexa 647, red) are shown. The white box in MERGE marks the magnified location (Zoom). White bar: 10 μm.

To corroborate that TvLEGU-1 and TvLEGU-2 were indeed secreted by metabolically active *T. vaginalis*, *in vitro* secretion kinetics were analyzed by the colocalization IFA of permeabilized parasites under GR and HG conditions using specific anti-legumain antibodies in combination with an anti-vesicle antibody (**Figures 3**–**6**) or between them (**Figures 7**, **8**). Thus, the absence of cross-reactivity between α-TvLEGU-1r and α-TvLEGU-2r antibodies was confirmed by WB. The α-TvLEGU-1r antibody bound only to the TvLEGU-1r antigen (**Supplementary Figure S1A**, lane 3) but not to the TvLEGU-2r antigen (**Supplementary Figure S1B**, lane 3). In contrast, the α-TvLEGU-2r antibody did not bind to the TvLEGU-1r antigen (**Supplementary Figure S1A**, lane 4). It was bound only to the TvLEGU-2r antigen (**Supplementary Figure S1B**, lane 4). The α-TvVes antibodies did not react with the recombinant legumains (see **Supplementary Figure S2A**). This lack of reactivity may be due to the absence of post-translational modifications (PTMs) or the presence of more immunogenic proteins in the contents of the vesicles used to produce the anti-TvVes antibody. In contrast, the α-TvLEGU-1r and α-TvLEGU-2r antibodies recognized different protein bands of varying sizes in the SPs (see **Supplementary Figure S2C**, lanes 9-12). These bands could correspond to TvLEGU-1 and TvLEGU-2 distinct processing steps previously reported (Rendón-Gandarilla et al., 2013; Euceda-Padilla et al., 2024) and have similar molecular weights to some of the protein bands detected by the α-TvVes antibody in the SPs (see **Supplementary Figure S2C**). Moreover, the α-TvVes antibody identified several proteins in the total protein extract (see **Supplementary Figure S2B**) and most of the proteins present in the SPs from parasites under GR and HG conditions, as expected (see **Supplementary Figure S2C**). Only a few differences were noted between the SPs from GR and HG conditions. Overall, these results demonstrate that each antibody specifically recognizes its respective legumain peptidase or the SPs found in vesicles.

### 3.4 TvLEGU-1 and TvLEGU-2 are independently secreted in vesicles and in a time-dependent manner under different glucose conditions

To demonstrate the active secretion of both legumains under different glucose conditions, we first performed colocalization IFA of each legumain with a vesicle marker. **Figures 3**, **4** show that TvLEGU-1 under GR and HG conditions colocalized with the vesicle marker at all three-time points. At 0 min, TvLEGU-1 colocalized with vesicles on the parasite membrane and only on some vesicles inside (**Figures 3l, r**, **4f, l**). At 30 min, a decrease in the whole fluorescence signal and the dotted mark of the vesicle marker was observed, suggesting active secretion. TvLEGU-1 was also detected in some vesicles but did not colocalize with the vesicle marker, mainly under HG conditions (**Figures 3x, ad**, **4r, x**). At 60 min of secretion, a greater reduction in the fluorescence signal from the vesicle marker and TvLEGU-1 under both glucose conditions and colocalization on the parasite surface (**Figures 3aj, ap**, **4ad, aj**) was observed mainly under HG conditions, confirming its secretion into the medium (**Figures 1**, **2**). These data were consistent with the WB results, in which a progressive reduction in the TvLEGU-1 fluorescence signal (**Figure 3**) corresponded to an increase in the amount of the TvLEGU-1 protein band in the SPs, mainly under HG conditions (**Figure 2C**).

**Figures 5**, **6** show that TvLEGU-2 under GR and HG conditions colocalized with the vesicle marker at all three time points. At 0 min, TvLEGU-2 colocalized with vesicles on the parasite membrane and only on some vesicles inside (**Figures 5**, **6f, l**). At 30 min, an increase in the dotted mark of the vesicle marker was observed under GR conditions, suggesting the formation of an increased number of vesicles at that time of secretion. The number of vesicles observed was greater in the HG than in the GR condition (**Figures 5**, **6r, x**). TvLEGU-2 also relocalized to these punctate areas of vesicles. At 60 min of secretion, a reduction in the fluorescence signal (FS) from the vesicle marker and TvLEGU-2 under both glucose conditions was observed, supporting its active secretion into the medium (**Figures 5**, **6ad, aj**). These data were consistent with the WB results, in which a progressive reduction in the TvLEGU-2 FS (**Figures 5**, **6**) corresponded to an increase in the amount of the TvLEGU-2 protein band in the SPs, mainly under GR conditions (**Figure 2D**). IFA images of permeabilized parasites revealed colocalization of TvLEGU-1 or TvLEGU-2 with the vesicle marker, with a Pearson’s coefficient above 0.9 under both glucose conditions for the two proteins (**Figures 3**–**6**), mainly on the parasite surface. The decrease in FS of both legumains during the secretion kinetics is also supported by the quantitative analysis of the FS intensity of both peptidases (see **Supplementary Figure S3**), as shown in **Figures 3** to **6**. This analysis included biological replicates, each with technical duplicates. For the quantitative assessment, we evaluated between 34 and 97 parasites at each time point and glucose condition (refer to **Supplementary Tables S1**, **S2**).

### 3.5 TvLEGU-1 is secreted earlier than TvLEGU-2

We also followed the secretion kinetics of both legumains concurrently under both glucose conditions. **Figures 7**, **8** show the IFA images of permeabilized parasites, which revealed reduced colocalization between TvLEGU-1 and TvLEGU-2 at all times tested, with a Pearson’s coefficient of ~0.47 under both glucose conditions (**Figures 7**, **8**). Additionally, TvLEGU-1 was secreted earlier than TvLEGU-2 under GR (**Figure 7**) compared with HG conditions (**Figure 8**), whereas TvLEGU-2 remained on the parasite surface after 60 min of secretion under GR conditions (**Figure 7**), suggesting that not all TvLEGU-2 on the surface was secreted.

### 3.6 TvLEGU-1 and TvLEGU-2 are independently secreted in specific regions of *T. vaginalis* surface

**Figure 9:**
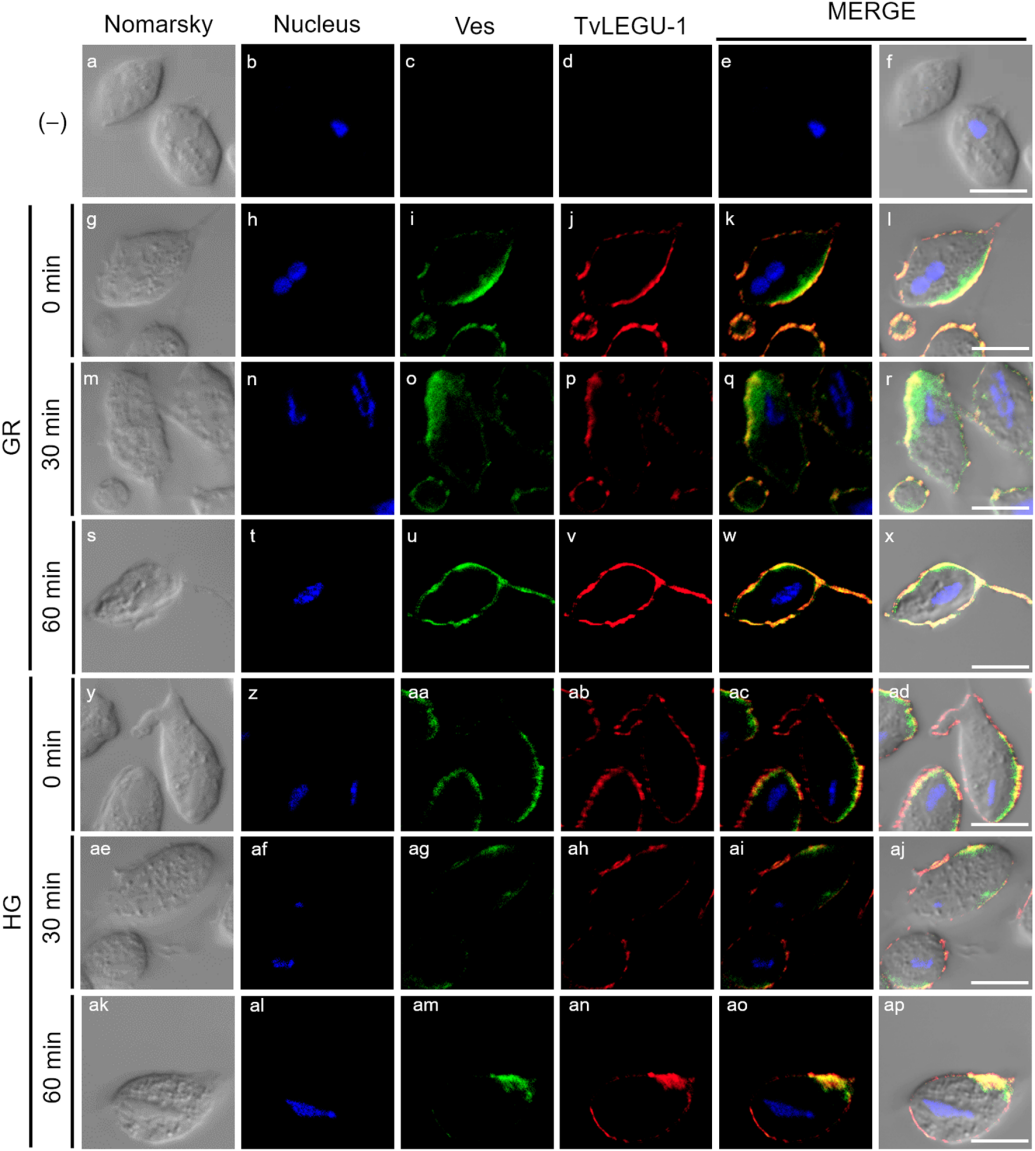
Secreted TvLEGU-1 polarizes to specific regions alongside vesicle markers in *T. vaginalis* under glucose restriction (GR) and high glucose (HG) conditions. Two-step IFA of parasites under GR **(g-x)** and HG **(y-ap)** conditions at different secretion times (0, 30, and 60 min) with Rα-TvLEGU-1r (1:300 dilution) and Mα-TvVes (1:100 dilution) antibodies to label vesicles. Negative control was performed by adding only the secondary antibody (-) **(a-f)**. Nuclei (DAPI, blue), vesicles (FITC, green), and TvLEGU-1 (Alexa 647, red) are shown. White bar: 10 μm.

**Figure 10:**
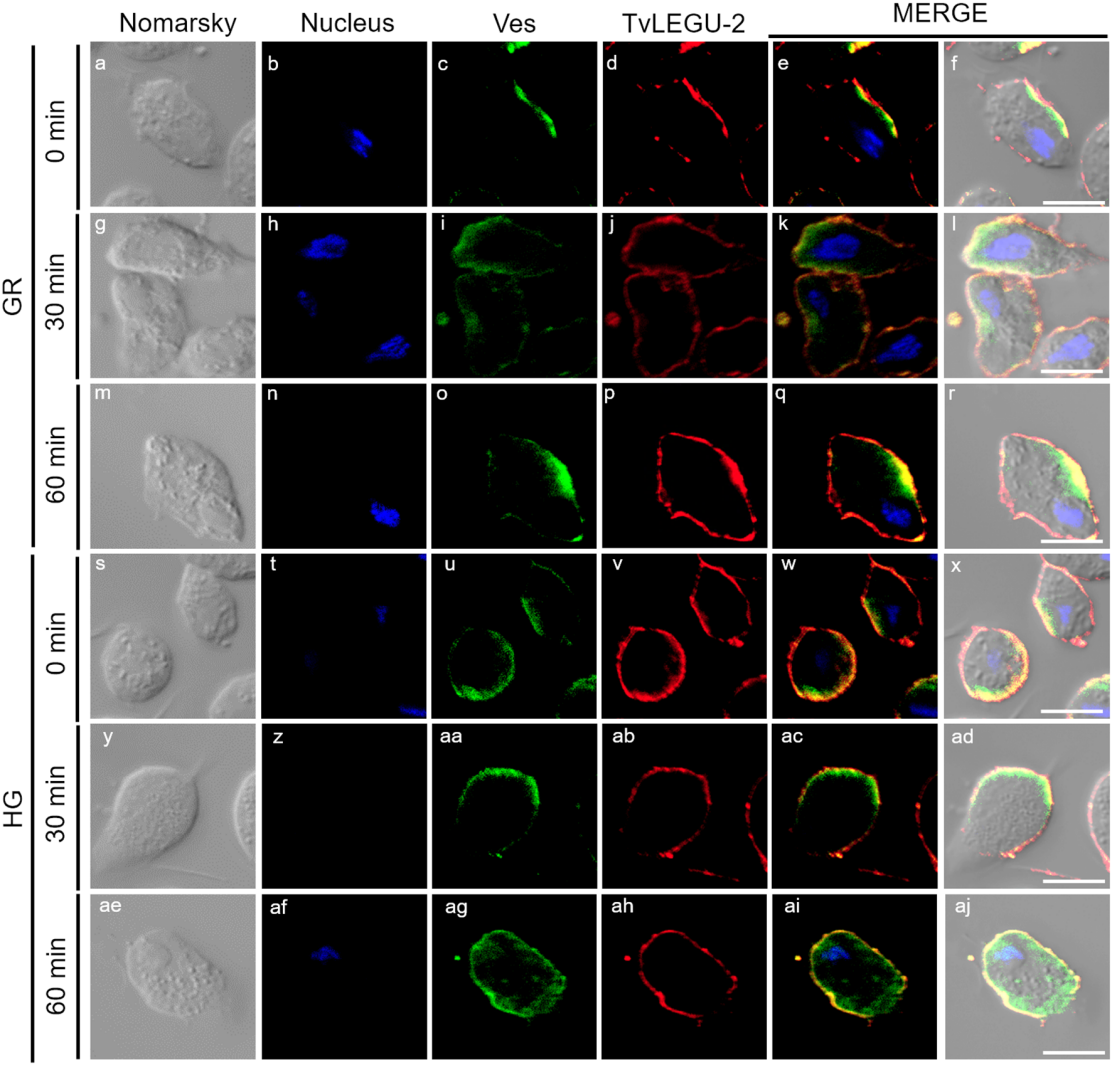
Secreted TvLEGU-2 polarizes to specific regions alongside vesicle markers in *T. vaginalis* under glucose restriction (GR) and high glucose (HG) conditions. Two-step IFA of parasites under GR **(a-r)** and HG **(s-aj)** at different secretion times (0, 30, and 60 min) with Rα-TvLEGU-2r (1:100 dilution) and Mα-TvVes (1:100 dilution) antibodies to label vesicles. Nuclei (DAPI, blue), vesicles (FITC, green), and TvLEGU-2 (Alexa 647, red) are shown. White bar: 10 μm.

We also analyzed the kinetic secretion of both legumains under GR and HG conditions (**Figures 9**, **10**) by double-pass IFA, in which the membrane was labeled first, followed by the cell interior. The results revealed that TvLEGU-1 and TvLEGU-2 colocalized with the vesicle marker in specific regions on the outer face of the parasite membrane at the three secretion time points and under both glucose conditions tested, with Pearson’s coefficients of 0.5 for GR and 0.6 for HG (**Figures 9**, **10**). Differences were observed at 60 min of secretion for TvLEGU-1 with the vesicle marker; under GR conditions colocalization was detected as a ring, whereas under HG conditions, colocalization was detected as capping-like polarization (**Figure 9**). In contrast, in TvLEGU-2 at 60 min, capping-like colocalization was detected under GR conditions, whereas ring colocalization was detected under HG conditions (**Figure 10**).

**Figure 11:**
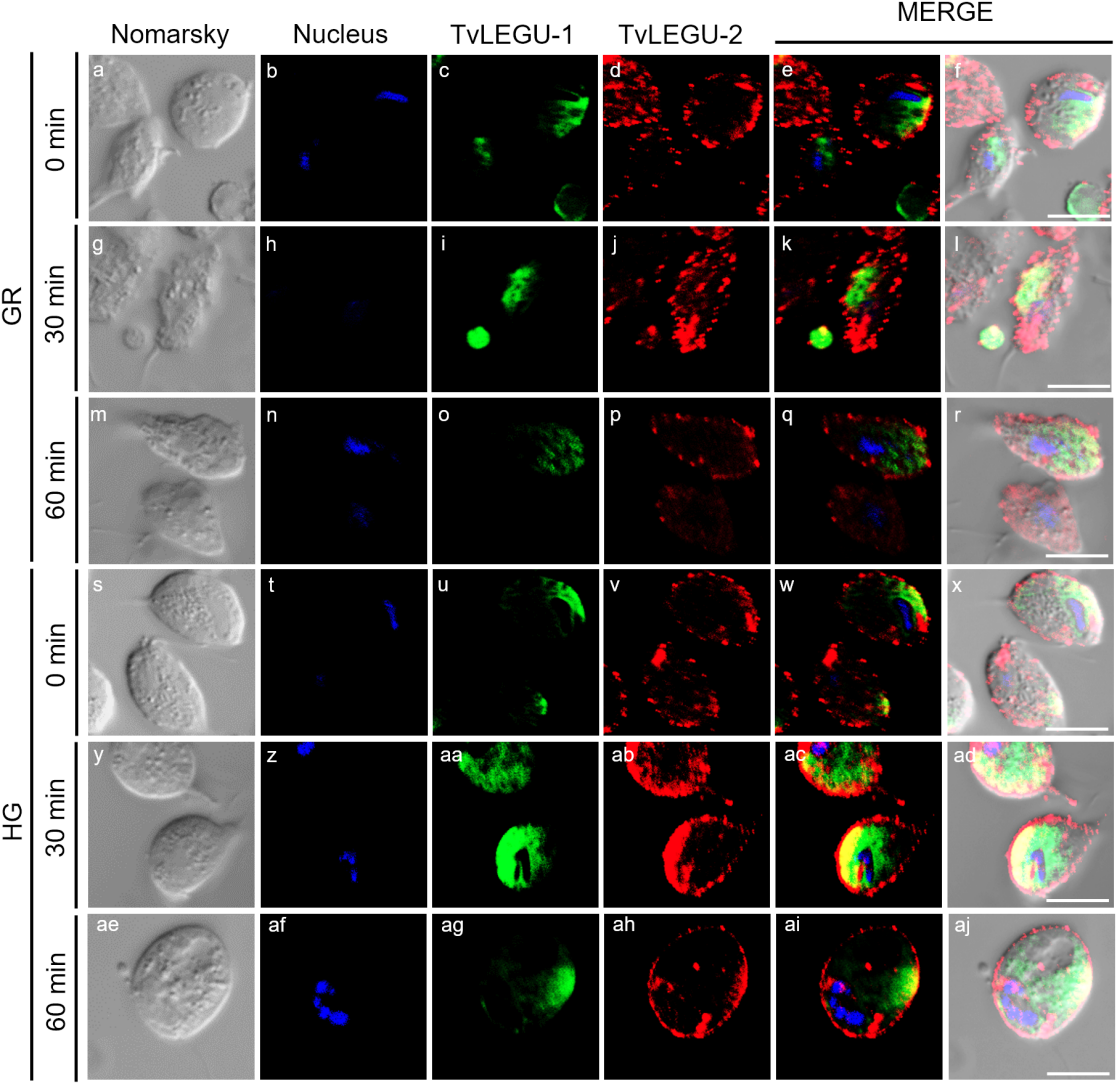
TvLEGU-1 and TvLEGU-2 polarize to specific regions in *T. vaginalis* during secretion under glucose restriction (GR) and high glucose (HG) conditions. Two-step IFA of parasites under GR **(a-r)** and HG **(s-aj)** conditions with Rα-TvLEGU-1r (1:300 dilution) and Mα-TvLEGU-2r (1:100 dilution) antibodies. Nuclei (DAPI, blue), TvLEGU-1 (FITC, green), and TvLEGU-2 (Alexa 647, red) are shown. White bar: 10 μm.

Furthermore, in colocalization kinetic assays of both legumains, the two molecules were observed to polarize to different regions of the parasites, with Pearson coefficient of ~0.2 under both glucose conditions (**Figure 11**). Interestingly, TvLEGU-2 appeared to cover most of the parasite membrane in vesicles, whereas TvLEGU-1 covered only certain parasite regions. These data revealed that both legumains were secreted in membrane-specific regions and that their secretion occurred mainly independently of each other.

### 3.7 TvLEGU-1 and TvLEGU-2 are secreted in different sizes and types of vesicles

We also analyzed the size and type of secreted vesicles containing TvLEGU-1 and/or TvLEGU-2 during the secretion kinetics by colocalization with the vesicle marker or between both legumains (**Supplementary Figure S4**). Vesicles containing TvLEGU-1 and/or TvLEGU-2 were observed before secretion (0 min), showing that secretion in *T. vaginalis* appeared active under basal conditions (**Supplementary Figure S4**). Vesicle sizes ranged from 0.5−5 μm under both glucose conditions, with similar sizes and proportions (**Supplementary Figures S4A, f-j, u-y**, **S4B, C, a-e, p-t**). Under the GR condition, the sizes detected were 0.5 μm (77.9%), 0.75 μm (6.9%), 1 μm (12.9%), 2 μm (1.9%), and 4-5 μm (0.3%); under the HG condition, they were 0.5 μm (84.3%), 0.75 μm (7.5%), 1 μm (6.9%), 2 μm (1%), and 4−5 μm (0.2%) (**Supplementary Figure S4D**). At 30 and 60 min, changes in the proportion of vesicles of different sizes ranging from 0.5−5 μm depending on the glucose condition were observed (**Supplementary Figures S4A, k-t, z-ai**; **S4B, C, f-o, u-ad**). At 30 min in the GR condition, the sizes of the vesicles detected were 0.5 μm (48.4%), 0.75 μm (8.2%), 1 μm (32.3%), 2 μm (9.7%), and 4–5 μm (1.4%); under the HG condition, they were 0.5 μm (69.4%), 0.75 μm (12.7%), 1 μm (15.5%), 2 μm (1.8%), and 4–5 μm (0.5%). At 60 min in the GR condition, the sizes of the vesicles detected were 0.5 μm (46.7%), 0.75 μm (10.5%), 1 μm (32.9%), 2 μm (7.9%), and 4–5 μm (2%); in the HG condition, they were 0.5 μm (55.9%), 0.75 μm (17%), 1 μm (23.9%), 2 μm (2.7%), and 4–5 μm (0.3%) (**Supplementary Figure S4D**). In addition, an increase in the number of vesicles was observed under both glucose conditions from 30 min of secretion (**Supplementary Figure S4D**) compared with the basal condition and exacerbated under the HG condition.

### 3.8 TvLEGU-1 and TvLEGU-2 are also secreted in vesicles protruding from axostyle, flagella, pseudopods, and pore-like structures

**Figure 12:**
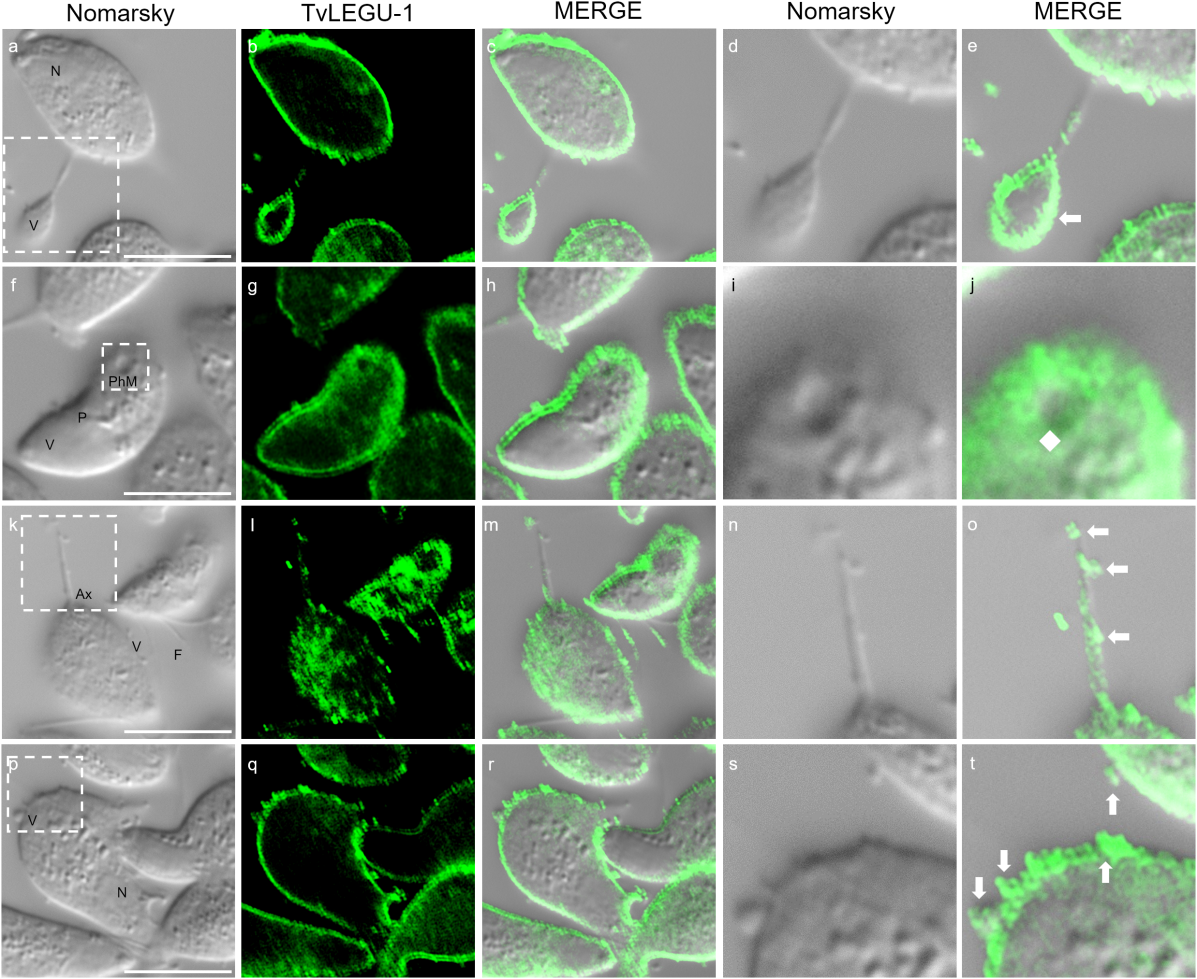
TvLEGU-1 is secreted through different membrane regions in *T. vaginalis*. IFA of permeabilized parasites at 30 min of secretion under HG conditions **(a-t)** with Rα-TvLEGU-1 antibody (1:300 dilution; FITC, green). White arrow: vesicle, white diamond: phagocytic mouth. V, vesicle; F, flagellum; N, nucleus; Ax, axostyle; PhM, phagocytic mouth. White bar: 10 μm. The white box marks the magnified location. White bar: 10 μm.

**Figure 13:**
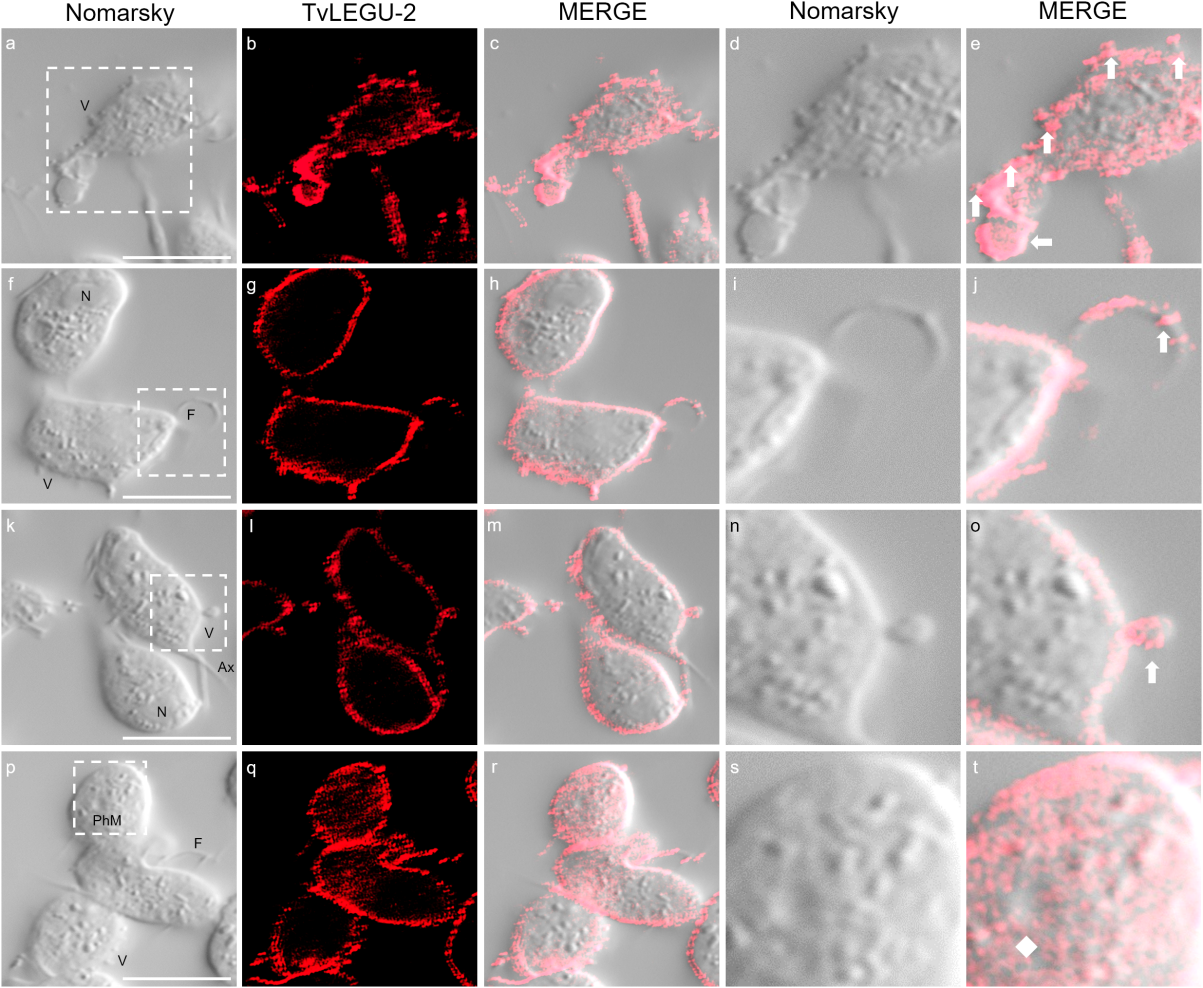
TvLEGU-2 is secreted through different membrane regions in *T. vaginalis*. IFA of permeabilized parasite after secretion at 30 min under HG conditions **(a-t)** with the Rα-TvLEGU-2r antibody (1:100 dilution; Alexa 647, red). White arrow: vesicle; white diamond: phagocytic mouth. V, vesicle; F, flagellum; N, nucleus; Ax, axostyle; PhM, phagocytic mouth. White bar: 10 μm. The white box marks the magnified location.

**Figure 14:**
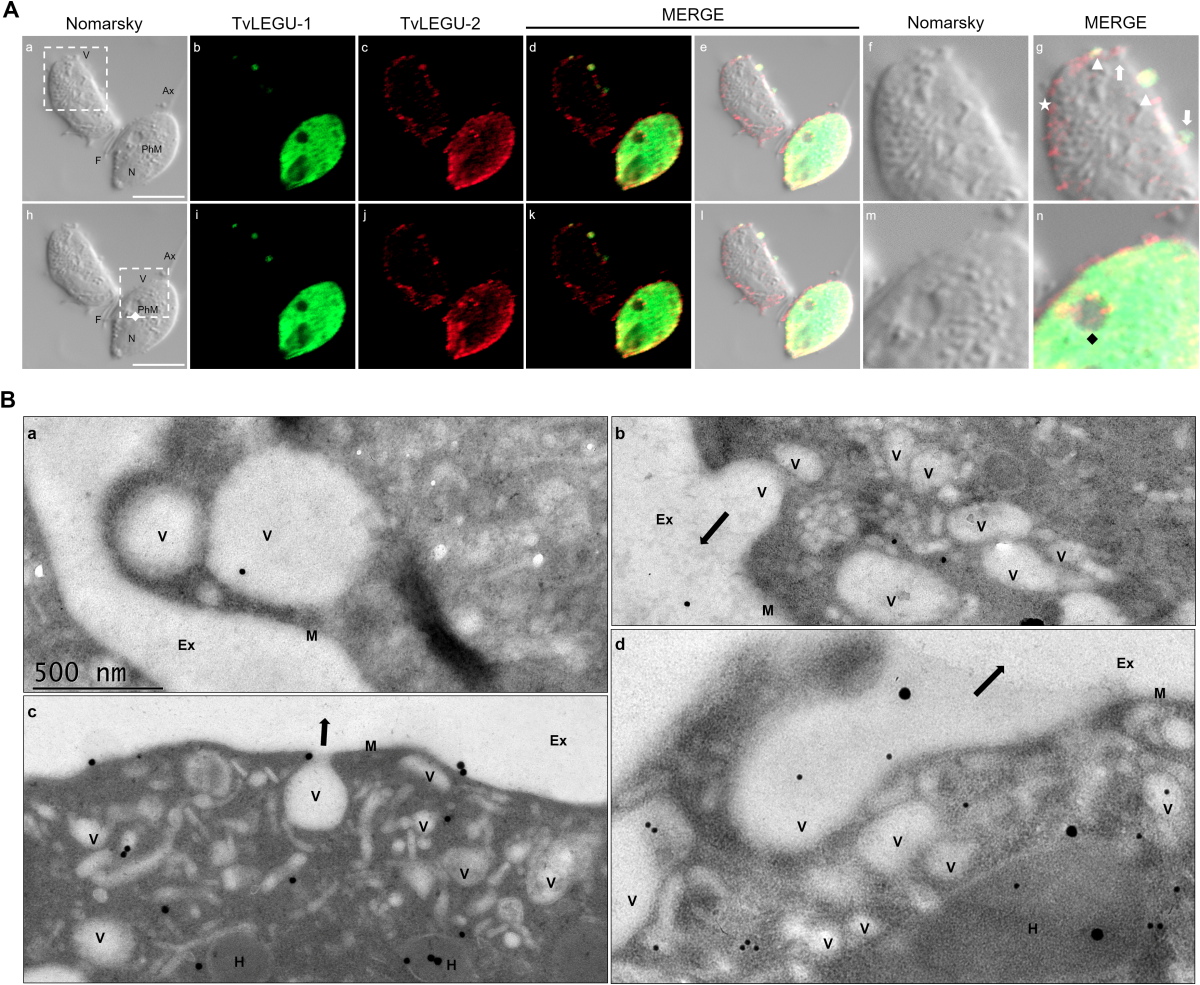
TvLEGU-1 and TvLEGU-2 are secreted through different membrane regions in *T. vaginalis*. **(A)** IFA colocalization of permeabilized parasite after secretion at 30 min under the HG condition **(a-n)** with Rα-TvLEGU-1r (1:300 dilution) and Mα-TvLEGU-2 (1:100 dilution) antibodies. TvLEGU-1 (FITC, green) and TvLEGU-2 (Alexa 647, red). White arrow: vesicle labeled with only one peptidase (TvLEGU-1 or TvLEGU-2); white arrowhead: vesicle with both peptidase labels (TvLEGU-1 and TvLEGU-2); white star: vesicle unlabeled with both peptidases (TvLEGU-1 and TvLEGU-2), black and white diamonds: phagocytic mouth. V: vesicle, F: flagellum, N: nucleus, Ax: axostyle, PhM: phagocytic mouth. White bar: 10 μm. The white box marks the magnified location. **(B)** Gold immunolocalization assay by TEM of parasites in GR **(a, b, d)** and HG **(c)** conditions with Mα-TvLEGU-2pep antibody (1:10 dilution). The 15-nm gold particle: TvLEGU-2 **(a, b)**, 30-nm gold particle: TvLEGU-2 **(c, d)**. V, vesicle; H, hydrogenosome; M, membrane; Ex, exterior. Black arrow: secretion to the outside of *T. vaginalis*.

**Figure 15:**
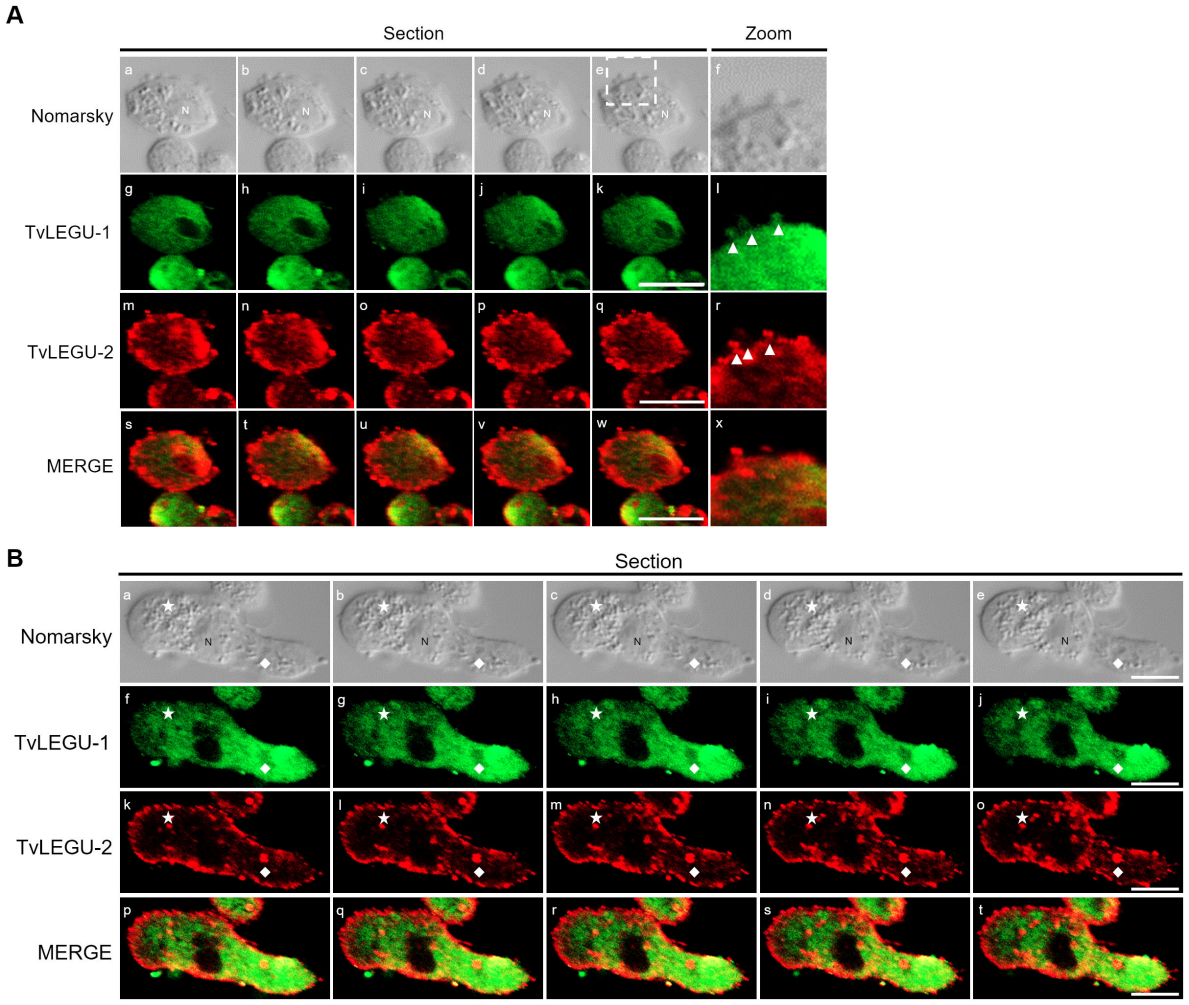
TvLEGU-1 and TvLEGU-2 are secreted in vesicles through different membrane regions in *T. vaginalis*. **(A)** IFA colocalization of parasites during the secretion process at 60 min in the HG condition **(a-x)** using rabbit (Rα-TvLEGU-1r; 1:300 dilution) **(g-l; s-x)** and mouse (Mα-TvLEGU-2r; 1:100 dilution) **(m-r; s-x)** antibodies. TvLEGU-1 (FITC, green) and TvLEGU-2 (Alexa 647, red). Arrowhead: vesicle. N, nucleus. The white box indicates the magnified location **(f, l, r, x)**. **(B)** IFA of parasites during the process of secretion at 60 min in the HG condition **(a-t)** using rabbit (Rα-TvLEGU-1r; 1:300 dilution) **(f-j; p-t)** and mouse (Mα-TvLEGU-2r; 1:100 dilution) **(k-o; p-t)** antibodies. TvLEGU-1 (FITC, green) and TvLEGU-2 (Alexa 647, red). Ax, axostyle; F, flagellum; N, nucleus. White diamond: phagocytic mouth; white star: release of vesicular contents to the exterior.

We observed that these localization patterns, relocalization, and secretion differed between TvLEGU-1 (**Figure 12**) and TvLEGU-2 (**Figure 13**). TvLEGU-1 was secreted earlier than TvLEGU-2 (**Figures 7**, **8**). Analysis of the secretion pattern by IFA and TEM revealed that both peptidases were actively secreted through different parasite membrane regions (82 and 91%, respectively) (**Figures 12**-**14**; **Supplementary Tables S3**, **S4**; **Supplementary Figures S5**, **S6**). They were secreted mainly in vesicles protruding from the cell membrane (80 and 74%, respectively) (**Supplementary Tables S3**, **S4**; **Supplementary Figure S6**). Some carried both peptidases as cargo, although not always in the same vesicles (**Figures 14**, **15**; **Supplementary Figure S4**). Others were transporting TvLEGU-1 and TvLEGU-2 from the parasite membrane. Those vesicles containing one or the other legumain also showed marks on the outside of the vesicle (**Supplementary Figure S4A**, panels h and m; **Supplementary Figure S4C, w**). Both legumain labels were also observed in a reduced proportion, in vesicles on the axostyle (14 and 16%, respectively), flagella (6 and 10%, respectively) (**Figures 12**, **13**; **Supplementary Figures S5**, **S6**; **Supplementary Tables S3**, **S4**), and pseudopods (**Figure 15B**), through pores on the plasma membrane (**Figure 14A**), and by the release of vesicular contents to the exterior by the fusion of vesicles with the cell membrane (**Figure 14B**; **Supplementary Figure S5**). These data revealed that *T. vaginalis* utilized different mechanisms and membrane regions to release proteins after stimulation, such as different glucose concentrations, since multiple different size vesicles and proportions containing both or individual legumain were observed by confocal and electron microscopy with specific anti-legumain antibodies (**Figures 12**-**15**; **Supplementary Tables S3**, **S4**; **Supplementary Figures S4**-**S6**).

## 4 Discussion

Despite the extensive *T. vaginalis* degradome, only nine CPs have been identified experimentally via mass spectrometry: seven cathepsin L-like (TvCP1, TvCP2, TvCP3, TvCP4, TvCP4-like, TvCP12, and TvCP39) and two asparaginyl endopeptidases of the C13 family (TvLEGU-1 and TvLEGU-2) (Ramón-Luing et al., 2010; Hernández et al., 2014; Arroyo et al., 2015; Rendón-Gandarilla et al., 2013; Euceda-Padilla et al., 2024). These CPs have been characterized as important virulence factors that participate in cytotoxicity, hemolysis, and cytoadherence (Arroyo et al., 2015; Hernández et al., 2014). Moreover, these CPs are highly regulated by environmental factors such as pH, and iron, glucose, zinc, and polyamine concentrations (Arroyo et al., 2015). During trichomoniasis, vaginal glucose levels fluctuate (Miranda-Ozuna et al., 2016), revealing the relevance of the glucose concentration in the vaginal environment to trichomonal cytotoxicity and the induction of host epithelial cell apoptosis, which are differentially modulated by glucose (Miranda-Ozuna et al., 2019).

This study revealed that TvLEGU-1 and TvLEGU-2 from *T. vaginalis* were actively secreted by the parasite regardless of the glucose concentration. The secretion products showed legumain proteolytic activity, consistent with the molecular weight of the most abundant protein bands (30 and 29 kDa, respectively) detected in the SPs, which corresponded to the active peptidase (**Figures 1**, **2**).

In addition to their main localization in the endolysosomal system, legumains are secreted into the extracellular medium and are suggested to have multiple extracellular functions, such as interactions with integrin receptors and extracellular matrix components to prolong their activity in acidic extracellular environments and to be associated with extracellular matrix remodeling, including fibronectin degradation or mesenchymal stem cell differentiation (Lunde et al., 2019). The peptidases TvLEGU-1 and TvLEGU-2 are the most abundant legumains in the *T. vaginalis* proteomes (Riestra et al., 2015; Stáfková et al., 2018; Nievas et al., 2018; Dias-Lopes et al., 2018; Zimmann et al., 2022). Both were detected in the endolysosomal system, such as the Golgi apparatus, ER, vesicles, cell membrane, hydrogenosomes, and lysosomes. We also detected them in vesicles protruding from the flagella and axostyle. TvLEGU-1 and TvLEGU-2 are secreted *in vitro* and during infection, making them important biomarkers for trichomoniasis (Ramón-Luing et al., 2010; Rendón-Gandarilla et al., 2013; Euceda-Padilla et al., 2024).

We investigated whether the TvLEGU-1 and TvLEGU-2 secreted by the parasite were proteolytically active. Interestingly, we observed that under both glucose conditions, legumain-like activity was reduced in the parasite extracts throughout the secretion period. The proteolytic activity of legumain increased in the SPs throughout the *in vitro* secretion time (**Figure 1**), which showed that the secreted legumains possessed proteolytic activity and were continuously secreted by the parasite in a timely manner. The proteolytic activity of TvLEGU-1 is necessary for the parasite cytoadherence process to occur. Thus, we speculate that in *T. vaginalis*, an activation cascade involving the participation of TvLEGU-1 activity on the parasite surface could occur to unmask parasite adhesins and activate the papain-like CPs involved in host protein degradation (Rendón-Gandarilla et al., 2013). Whether active TvLEGU-2 is involved in any of the pathogenic mechanisms of *T. vaginalis* remains unclear, and studies are in progress to solve this problem. WB assays (**Figure 2**) and IFA (**Figures 3**-**8**) revealed that TvLEGU-1 and TvLEGU-2 were secreted in a time-dependent manner, with the mature peptidase being secreted in greater amounts at 60 min (**Figure 2**), consistent with the proteolytic activity results showing that the secretion had legumain activity (**Figure 1**). These results suggested that both peptidases were responsible for this activity. The secretion of mature human legumain (HsLMN) has been linked to carotid atherosclerosis (Lunde et al., 2017b). However, the roles of the secreted zymogen and mature TvLEGU-1 and TvLEGU-2 peptidases in the pathogenesis of *T. vaginalis* are not yet known and require further study.

We also analyzed the possible TvLEGU-1 and TvLEGU-2 secretion mechanisms. IFA assays revealed that TvLEGU-1 and TvLEGU-2 were secreted from the parasite at different times in multiple membrane regions. Interestingly, both legumains polarized in parasite-specific regions (**Figures 9**-**11**), suggesting a mechanism of legumain localization that might be regulated by glycosylation. In humans, glycosylation of legumains is required for the secretion and reentry of HsLMN into the cell (Lunde et al., 2017a). However, only the phosphorylation and glycosylation of TvLEGU-1 have been demonstrated by WB analysis (Rendón-Gandarilla et al., 2013 and our unpublished data). *In silico* analyses of TvLEGU-2 suggested that it could be glycosylated, which could be supported by its localization in the Golgi complex (Euceda-Padilla et al., 2024), as has also been previously shown for TvLEGU-1 (Rendón-Gandarilla et al., 2013). Therefore, we propose that differential glycosylation could modulate the secretion of both trichomonad legumains.

TvLEGU-1 and TvLEGU-2 exhibited different secretion patterns, both were secreted in vesicles protruding from the plasma membrane, with some carrying both peptidases and others carrying either TvLEGU-1 or TvLEGU-2 (**Figures 12**-**15**; **Supplementary Figures S4**, **S5**); in vesicles from the axostyle and flagella (**Figures 12**, **13**, **15**; **Supplementary Tables S3**, **S4**, **Supplementary Figures S4**-**S6**), through pores in the plasma membrane (**Figure 14A**); and by release of the vesicle contents by fusion of the vesicles with the cell membrane (**Figure 14B**). These data suggested that TvLEGU-1 and TvLEGU-2 were secreted by the classical secretion pathway via passage through the Golgi apparatus to reach the plasma membrane and released to the exterior in vesicles fused with the plasma membrane. These data also suggest that unconventional secretion pathways may occur for these two proteins, in which their release to the outside from trichomonads might involve type I (mediated by membrane pore formation) and type III (mediated by membrane-bound organelles that deviate from their normal function and become secretory, e.g., vesicles of the endolysosomal system and autophagosomes) secretion pathways (Rabouille, 2017). Unconventional secretion of peptidases from *T. vaginalis* by release of lysosomal contents to the outside, as occurs with TvCP2, has recently been demonstrated (Zimmann et al., 2022). Given that the legumains TvLEGU-1 and TvLEGU-2 are peptidases that localize in lysosomes (Rendón-Gandarilla et al., 2013; Euceda-Padilla et al., 2024), we cannot rule out that the same unconventional secretory pathway could also occur for these two peptidases.

The secretion of peptidases by *T. vaginalis* appears to be a dynamic process, in which peptidases such as TvLEGU-1 and TvLEGU-2 are secreted by the release of extracellular vesicles protruding from the plasma membrane, axostyle, and flagella in different regions of the parasite simultaneously, either separately or in combination (**Figures 12**-**15**; **Supplementary Figures S4**-**S6**). Our findings are supported by recent characterizations of secretion in *T. vaginalis*, which reveal a highly dynamic process with vesicles protruding from multiple areas of the parasite, carrying diverse contents—including TvLEGU-1 and TvLEGU-2 peptidases (Nievas et al., 2018; Twu et al., 2013; Rada et al., 2019; and our unpublished results). Notably, Nievas et al. (2018) and Coceres et al. (2021) reported the secretion of extracellular vesicles through parasite flagella. Furthermore, Sudhakar et al. (2021) recently identified TvLEGU-1 among other peptidases in the proteome of isolated flagella from *T. vaginalis*. These data reinforce our observation of the FS of TvLEGU-1 and TvLEGU-2 in flagella and vesicles protruding from them. However, further studies are necessary to confirm the localization of TvLEGU-1 and TvLEGU-2 in the flagella of *T. vaginalis*. These data suggest that both peptidases might play key roles in the pathogenicity of *T. vaginalis*; further analyses should be performed to determine their roles. Work is underway to address these issues.

## 5 Conclusions

In conclusion, the peptidases TvLEGU-1 and TvLEGU-2 are actively secreted by the parasite as zymogens and mature peptidases, the latter with proteolytic activity. In addition, both legumains are differentially secreted by *T. vaginalis*, polarizing in different regions (**Figures 9**-**11**), and secreted simultaneously and independently (**Figures 14**, **15**). The role of TvLEGU-2 and the secreted TvLEGU-1 and TvLEGU-2 peptidases in the pathogenesis of *T. vaginalis* remains to be analyzed.

## Data Availability

The original contributions presented in the study are included in the article/**Supplementary Material**. Further inquiries can be directed to the corresponding author.
