# Supplementary material for: TvLEGU-1 and TvLEGU-2 biomarkers for trichomoniasis are legumain-like cysteine peptidases secreted in vitro in a time-dependent manner
Source: Front Parasitol. 2025 Mar 5;4:1546468. doi: 10.3389/fpara.2025.1546468 (PMC11920906; doi:10.3389/fpara.2025.1546468)
Supplement: Supplementary file 1 [file DataSheet1.pdf]

Supplementary Material

1.1 Supplementary Figures

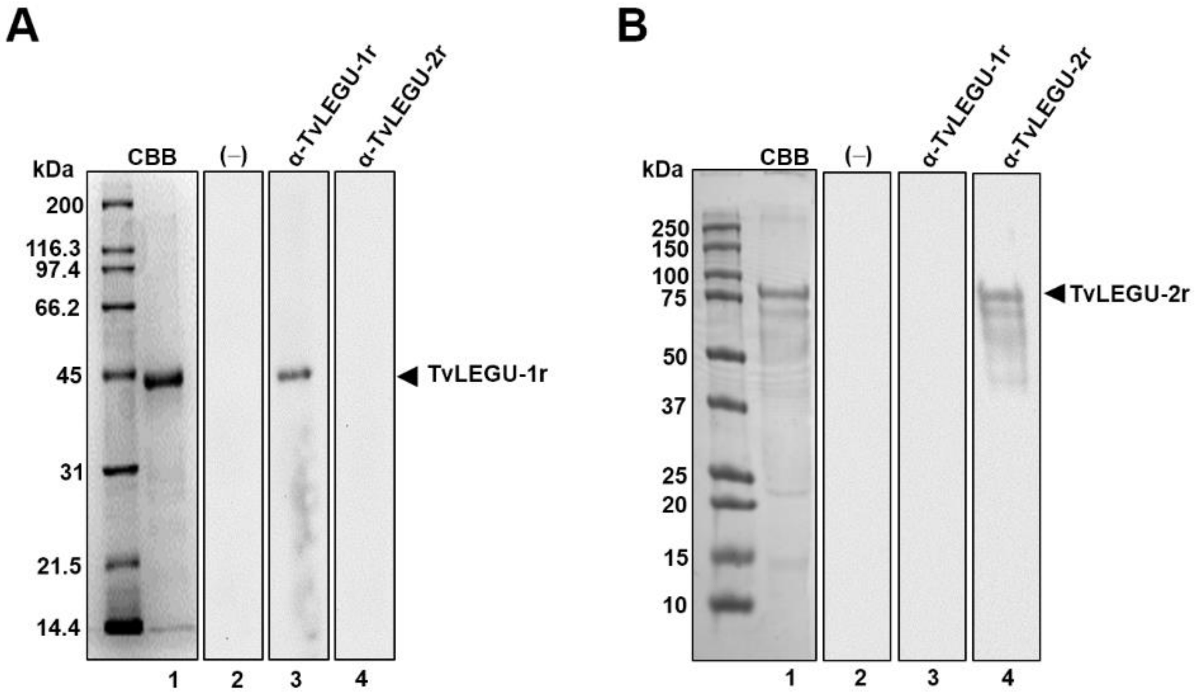

Supplementary Figure S1. Specificity of the α-TvLEGU-1 and α-TvLEGU-2 antibodies. (A) Purified fusion recombinant TvLEGU-1r protein with an expected size of ~46 kDa contained a 6-His tag (Ramón-Luing et al., 2010) analyzed by SDS–PAGE on 10% polyacrylamide gels and by WB assays of proteins transferred onto NC membranes (lanes 2-4) or Coomassie brilliant blue-stained (CBB; lane 1). WB assays of TvLEGU-1r incubated with Rα-TvLEGU-1r antibody (1:3000 dilution; lane 3), Rα-TvLEGU-2r antibody (1:1000 dilution; lane 4), or only with the secondary antibody as a negative control (-) (lane 2). The arrowhead points to the recombinant TvLEGU-1r protein band (~46 kDa). **(B)** Purified fusion recombinant TvLEGU-2r protein (~43 kDa) with an expected size of ~85 kDa contained a maltose-binding protein (MBP of ~42 kDa) tag (Euceda-Padilla et al., 2024) analyzed by SDS–PAGE on 10% polyacrylamide gels and by WB assays of proteins transferred onto NC membranes (lanes 2-4) or Coomassie brilliant blue-stained (CBB; lane 1). WB assays of TvLEGU-2r incubated with Rα-TvLEGU-2r antibody (1:1000 dilution; lane 4), Rα-TvLEGU-1r antibody (1:3000 dilution; lane 3), or only with the secondary antibody as a negative control (-) (lane 2). The arrowhead denotes the recombinant TvLEGU-2r (~85 kDa) protein band. kDa, molecular weight markers in kilodaltons (Bio-Rad).

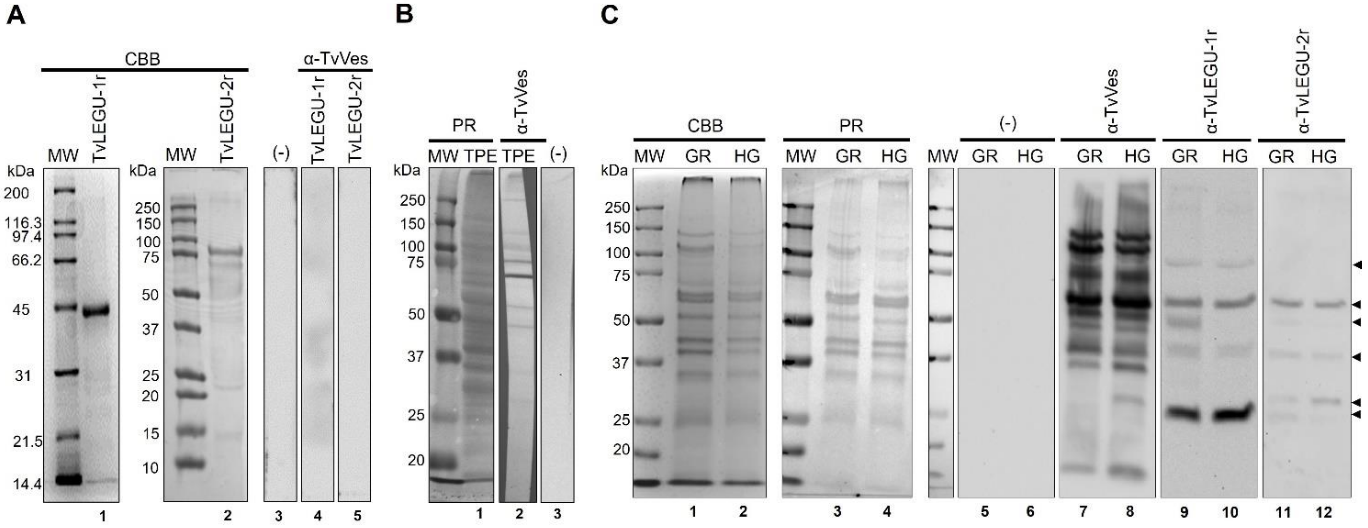

Supplementary Figure S2. Specificity of the α-TvVes antibody. (A) Purified fusion recombinant TvLEGU-1r protein with an expected size of ~46 kDa contained a 6-His tag (Ramón-Luing et al., 2010) (lane 1) and purified fusion recombinant TvLEGU-2r protein (~43 kDa) with an expected size of ~85 kDa contained a maltose-binding protein (MBP of ~42 kDa) tag (Euceda-Padilla et al., 2024) (lane 2) analyzed by SDS-PAGE on 10% polyacrylamide gels and by WB assays of proteins transferred onto NC membranes or Coomassie brilliant blue-stained (CBB; lane 1, 2). WB assays of TvLEGU-1r and TvLEGU-2r incubated with the α-TvVes antibody (1:1000 dilution) (lanes 4 and 5, respectively), or only with the secondary antibody as a negative control (-) (lane 3). MW, molecular weight markers in kilodaltons (Bio-Rad). **(B)** Total protein extracts **(**TPE) (lanes 1-3) analyzed by SDS-PAGE on 10% polyacrylamide gels and by WB assays of proteins transferred onto NC membranes Ponceau red-stained (PR; lane 1) or incubated with the α-TvVes antibody (1:1000 dilution), or only with the secondary antibody as a negative control (-) (lane 3). MW, molecular weight markers in kilodaltons (Bio-Rad). **(C)** Recognition of the α-TvVes antibody in secretion products obtain from parasites under GR and HG conditions. Protein profile of Coomassie Brilliant Blue-stained (CBB) secretion products (SPs) of parasites grown under GR and HG conditions (lanes 1 and 2, respectively). For WB assays, SPs from parasites incubated under GR and HG conditions were separated on 10% SDS-PAGE gels, transferred onto NC membranes and Ponceau red-stained (PR) (lanes 3 and 4, respectively). The NC membranes were incubated with the α-TvVes (1:1000 dilution) antibody to detect proteins recognized in the SPs (lanes 7 and 8) and with the α-TvLEGU-1r (lanes 9 and 10) and α-TvLEGU-2r (lanes 11 and 12) antibodies to detect the presence of TvLEGU-1 and TvLEGU-2 in the SPs. A duplicate NC membrane incubated only with the secondary antibody (-) (lanes 5 and 6) was used as a negative control. MW, molecular weight markers in kilodaltons (Bio-Rad). Arrowheads: despite position of protein bands recognized by the primary antibodies used.

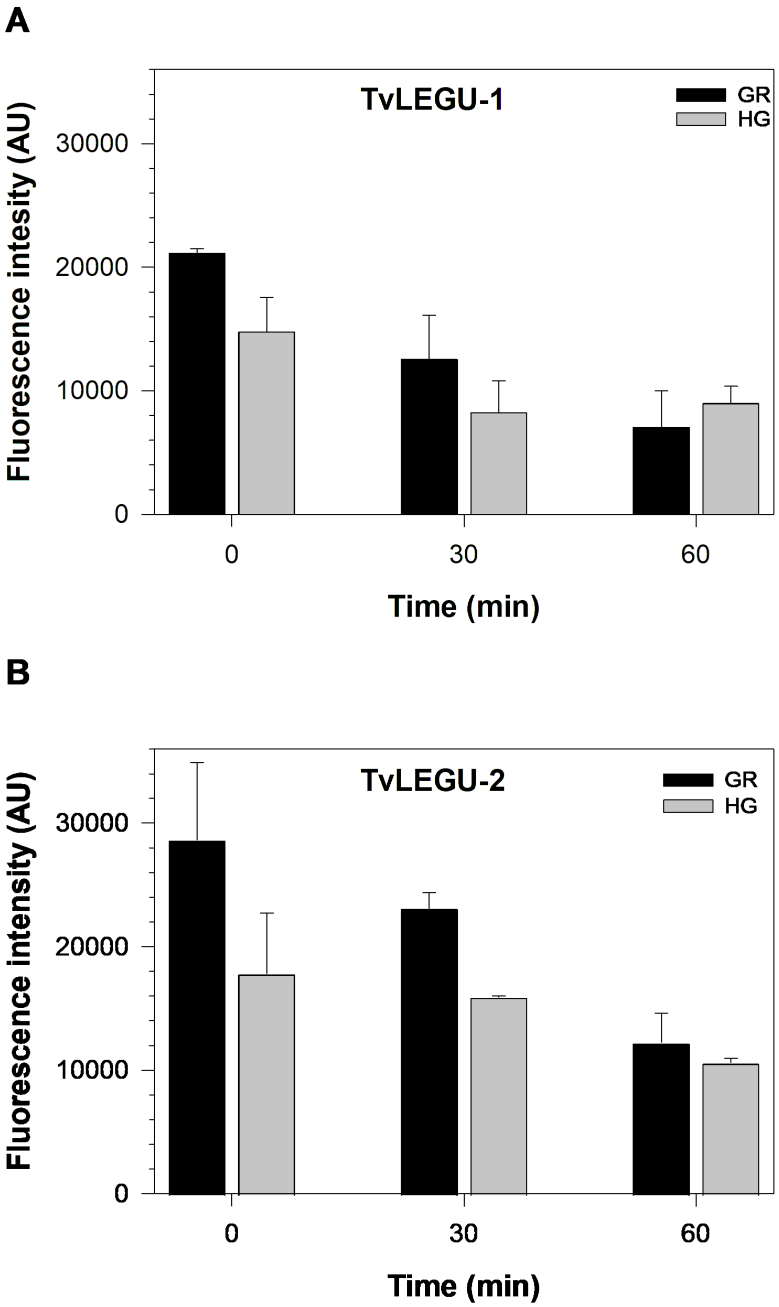

Supplementary Figure S3. Quantitative analysis of fluorescence intensity for TvLEGU-1 and TvLEGU-2 secretion kinetics under varying glucose conditions. (A) The quantitative analysis of parasite fluorescence intensity in arbitrary units (AU) for TvLEGU-1 under glucose-restriction (GR) and high-glucose (HG) conditions is illustrated in Figures 3 and 4, respectively. **(B)** The fluorescence intensity in AU of parasites for TvLEGU-2 under GR and HG conditions is shown in Figures 5 and 6, respectively. Data analysis and graphs were performed using Sigma-Plot 14.5. A summary of the number of parasites analyzed across four images for each time point from two independent experiments —each conducted in duplicate for TvLEGU-1 and TvLEGU-2— can be found in Supplementary Tables S1 and S2, respectively.

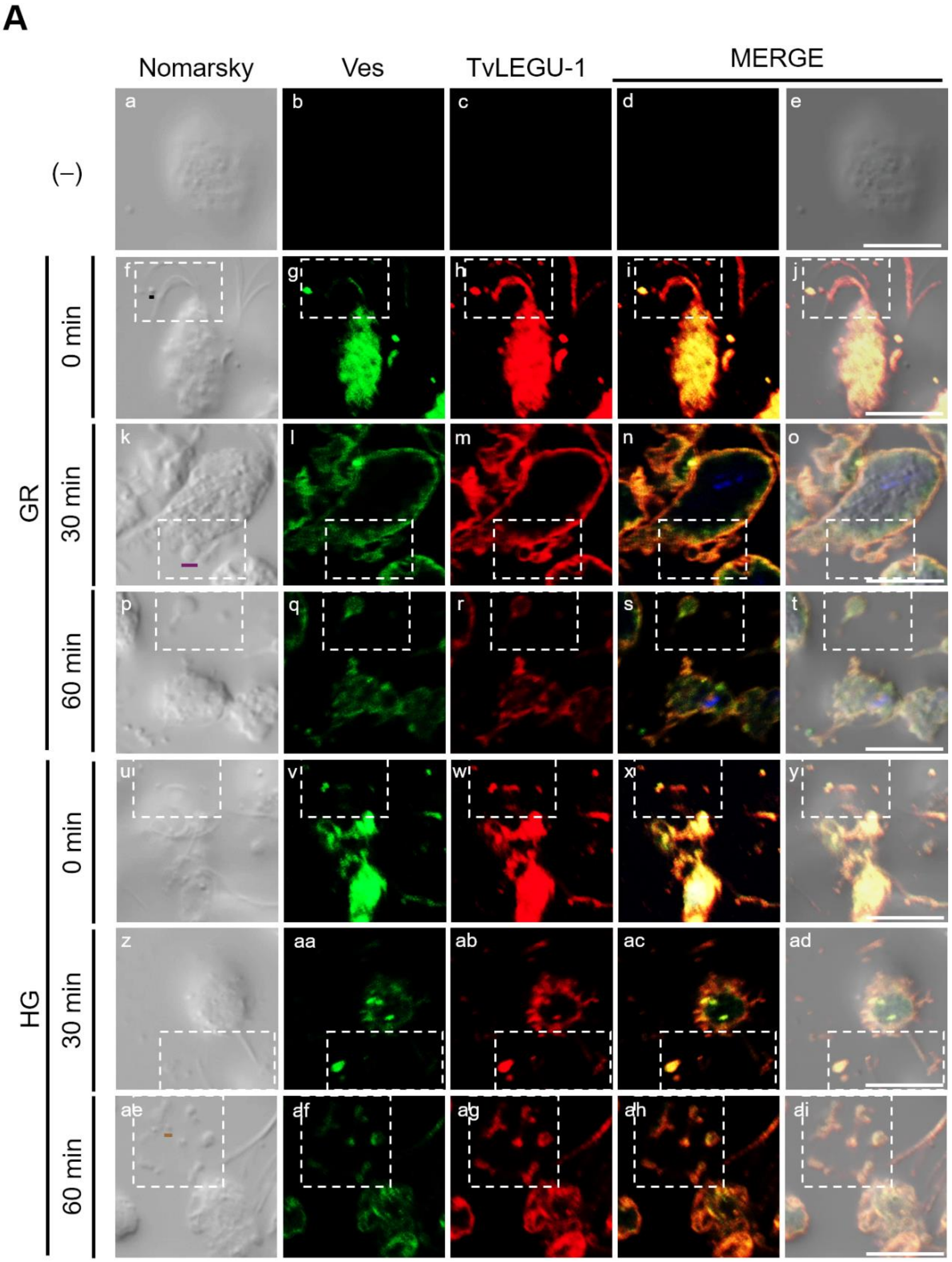
A
Nomarsky
Ves
TvLEGU-1
MERGE
(–)
GR
HG
0 min
30 min
60 min
0 min
30 min
60 min
a
b
c
d
e
f
g
h
i
j
k
l
m
n
o
p
q
r
s
t
u
v
w
x
y
z
aa
ab
ac
ad
ae
af
ag
ah
ai

B

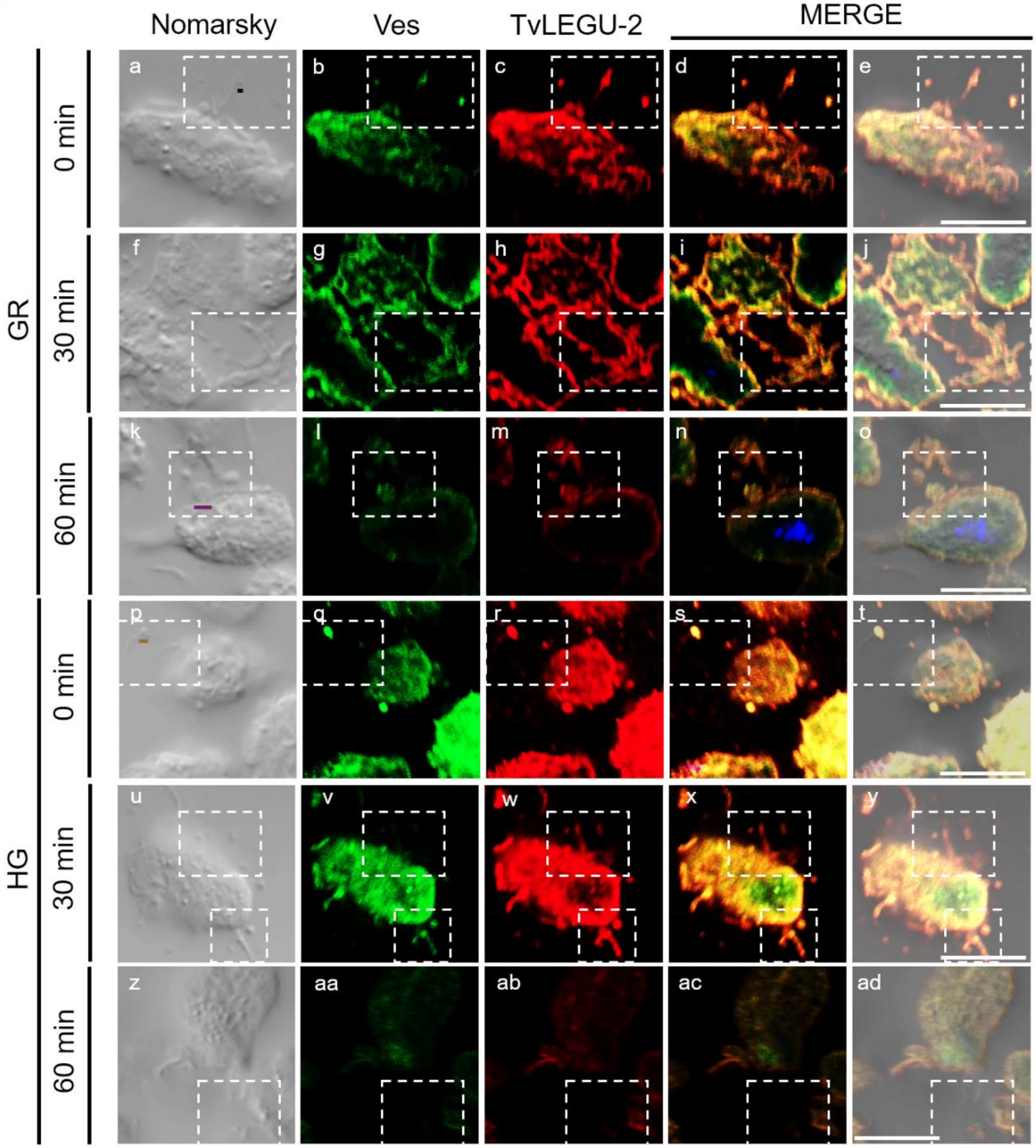
Nomarsky
Ves
TvLEGU-2
MERGE
GR
HG
0 min
30 min
60 min
0 min
30 min
60 min
a
b
c
d
e
f
g
h
i
j
k
l
m
n
o
p
q
r
s
t
u
v
w
x
y
z
aa
ab
ac
ad

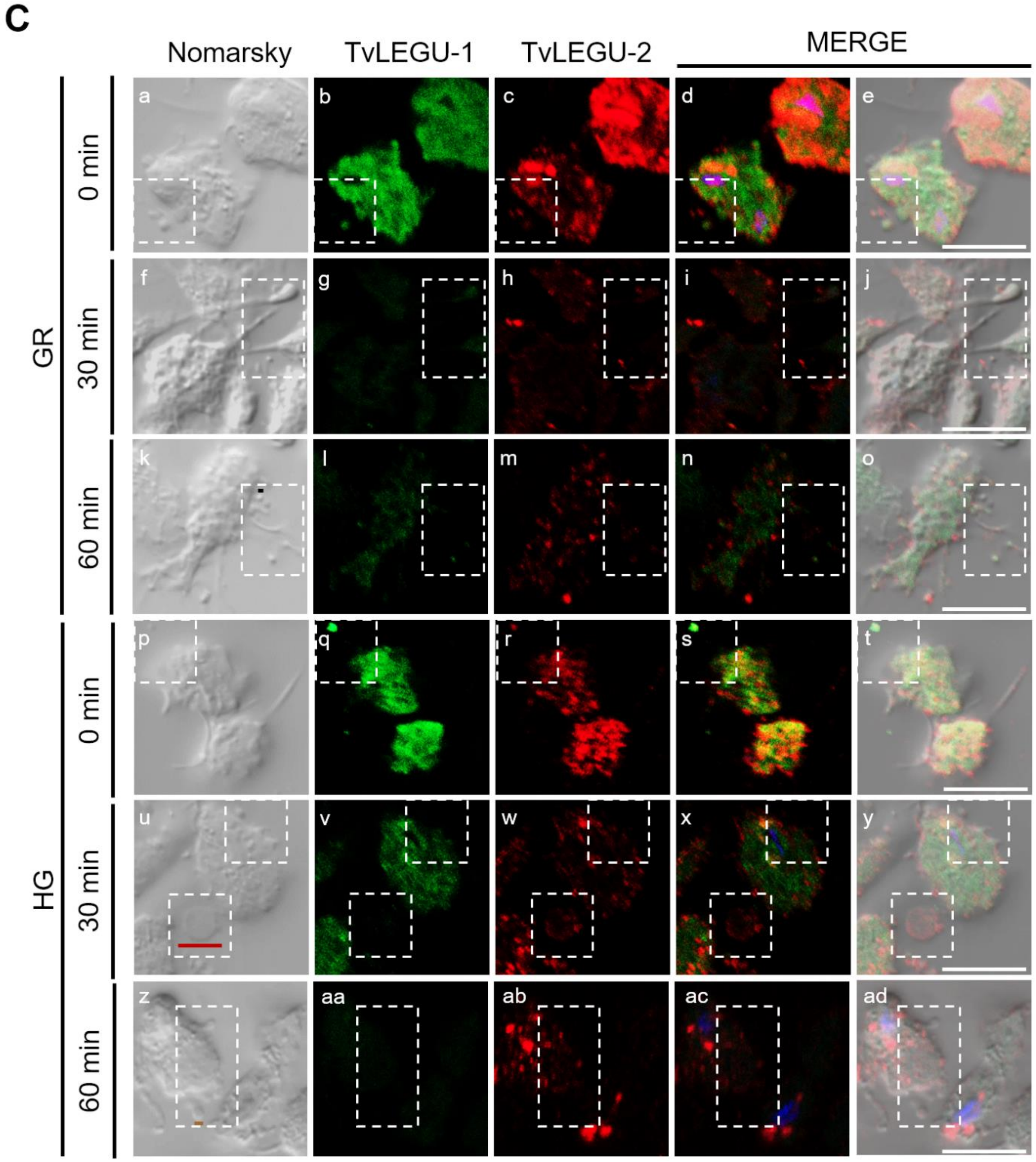
C
Nomarsky
TvLEGU-1
TvLEGU-2
MERGE
GR
0 min
30 min
60 min
HG
0 min
30 min
60 min
a
b
c
d
e
f
g
h
i
j
k
l
m
n
o
p
q
r
s
t
u
v
w
x
y
z
aa
ab
ac
ad

D

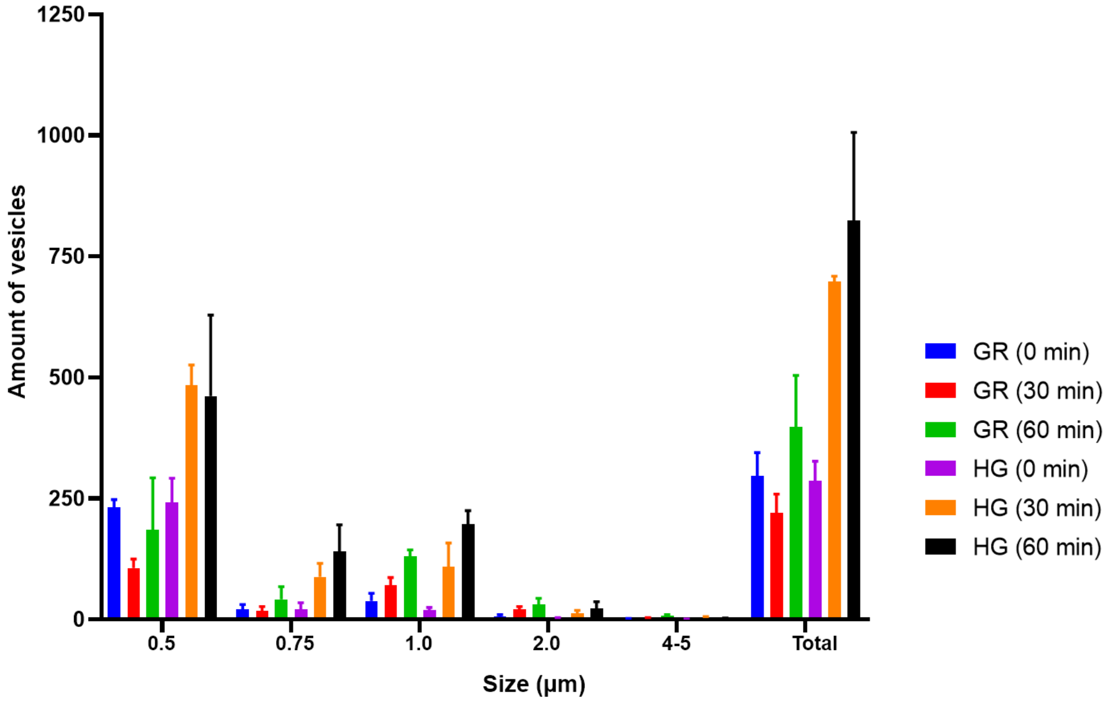

Supplementary Figure S4. Visualization of different sizes and proportions of TvLEGU-1- and TvLEGU-2-tagged vesicles in *T. vaginalis* under varying glucose conditions. (A) IFA of permeabilized parasites during the process of secretion under GR (f-t) and HG (u-ai) conditions in the secretion kinetic under GR conditions for 0 (f-j), 30 (k-o), and 60 min (p-t) and under HG conditions for 0 (u-y), 30 (z-ad), and 60 min (ae-ai) using Rα-TvLEGU-1r (1:300 dilution; Alexa 647, red) and Mα-TvVes (1:100 dilution; FITC, green) antibodies to label the surface and vesicles, respectively; nuclei (DAPI, blue). Only the secondary antibody was added (-) (a-e) for negative control. Black bar: 0.5 µm, brown bar: 1 µm, blue bar: 2 µm. White box: area of interest. **(B)** IFA of the secretion process in permeabilized parasites in the GR (a-o) and HG (p-ad) conditions based on the secretion kinetics under GR conditions at 0 (a-e), 30 (f-j), and 60 min (k-o) and under HG conditions at 0 (p-t), 30 (u-y), and 60 min (z-ad) using Rα-TvLEGU-2r (1:100 dilution; Alexa 647, red) and Mα-TvVes (1:100 dilution; FITC, green) antibodies to label the surface and vesicles, respectively; nuclei (DAPI, blue). Black bar: 0.5 µm, brown bar: 1 µm, blue bar: 2 µm. White box: area of interest. **(C)** IFA of permeabilized parasites during the secretion process under GR (a-o) and HG (p-ad) conditions based on secretion kinetics under GR conditions for 0 (a-e), 30 (f-j), and 60 min (k-o) and HG conditions at 0 (p-t), 30 (u-y), and 60 min (z-ad) using Rα-TvLEGU-1r (1:300 dilution; FITC, green) and Mα-TvLEGU-2r antibodies (1:100 dilution; Alexa 647, red), nuclei (DAPI, blue). Black bar: 0.5 µm, brown bar: 1 µm, red bar: 4–5 µm. White box: area of interest. **(D)** Analysis of the number of vesicles of different sizes in parasites under GR and HG conditions at 0, 30, and 60 min of secretion from two independent experiments, two images in each experiment. The analysis was performed with the Zeiss Zen 3.9 and Graph Pad Prism 8.0.1.

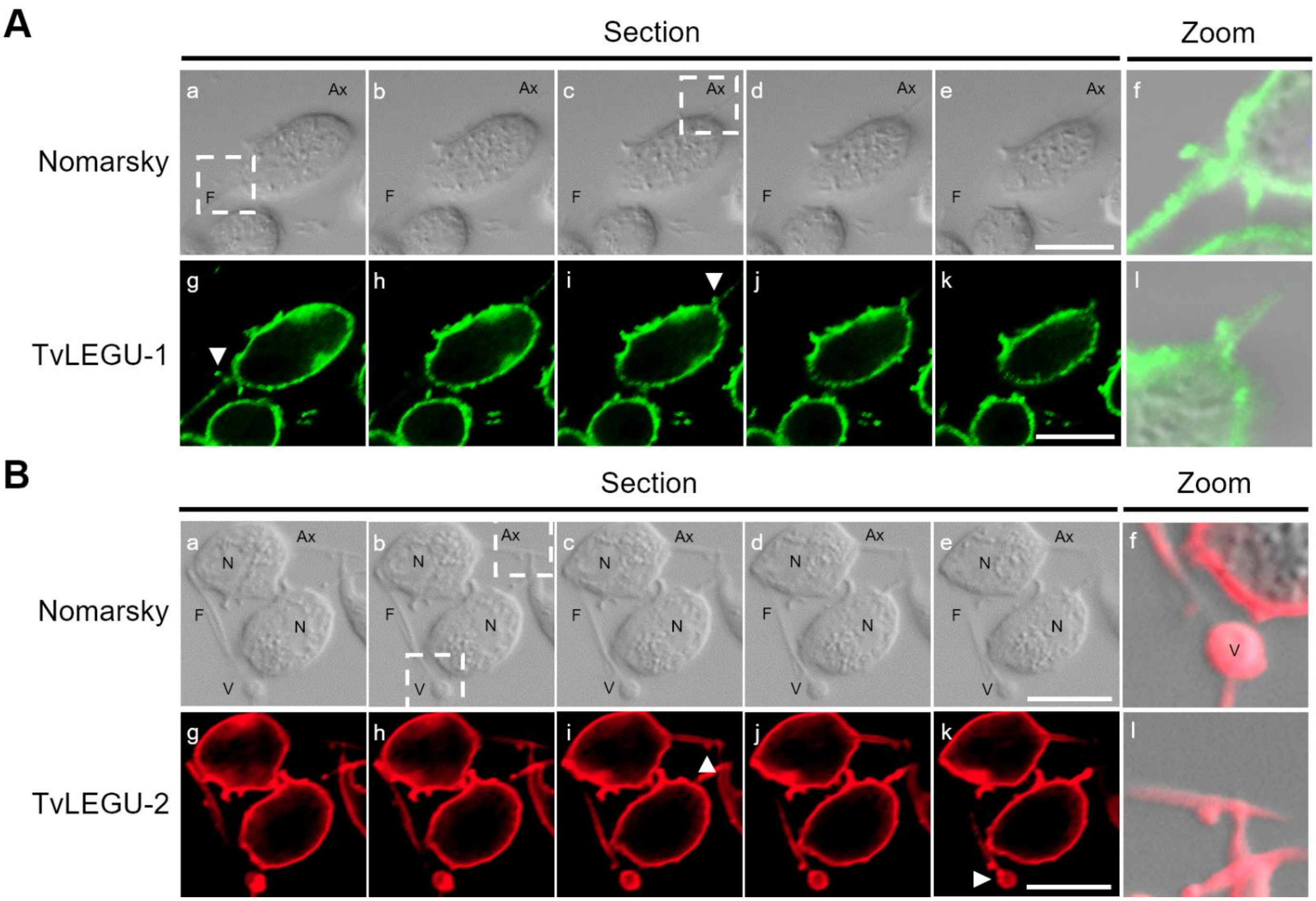

Supplementary Figure S5. TvLEGU-1 and TvLEGU-2 peptidases are secreted through different membrane regions in *T. vaginalis*. (A) IFA of parasites at 60 min of secretion under HG conditions using the Rα-TvLEGU-1r antibody (a-l) (1:300 dilution; FITC, green). Several optical sections are shown (a-l). White arrowhead: vesicle, F: flagellum, N: nucleus, Ax: axostyle. The white arrowheads indicate vesicles on the axostile. White bar: 10 µm. The white box denotes the magnified location (f, l). **(B)** IFA of parasite secretion after 30 min under HG conditions using the Rα-TvLEGU-2 antibody (a-l) (1:100 dilution; Alexa 647, red). Several optical sections are shown (a-l). White arrow: vesicle, F: flagellum, Ax: axostyle, V: vesicles. White arrowheads, vesicles on the axostile. White bar: 10 µm. The white box denotes the magnified location (f, l).

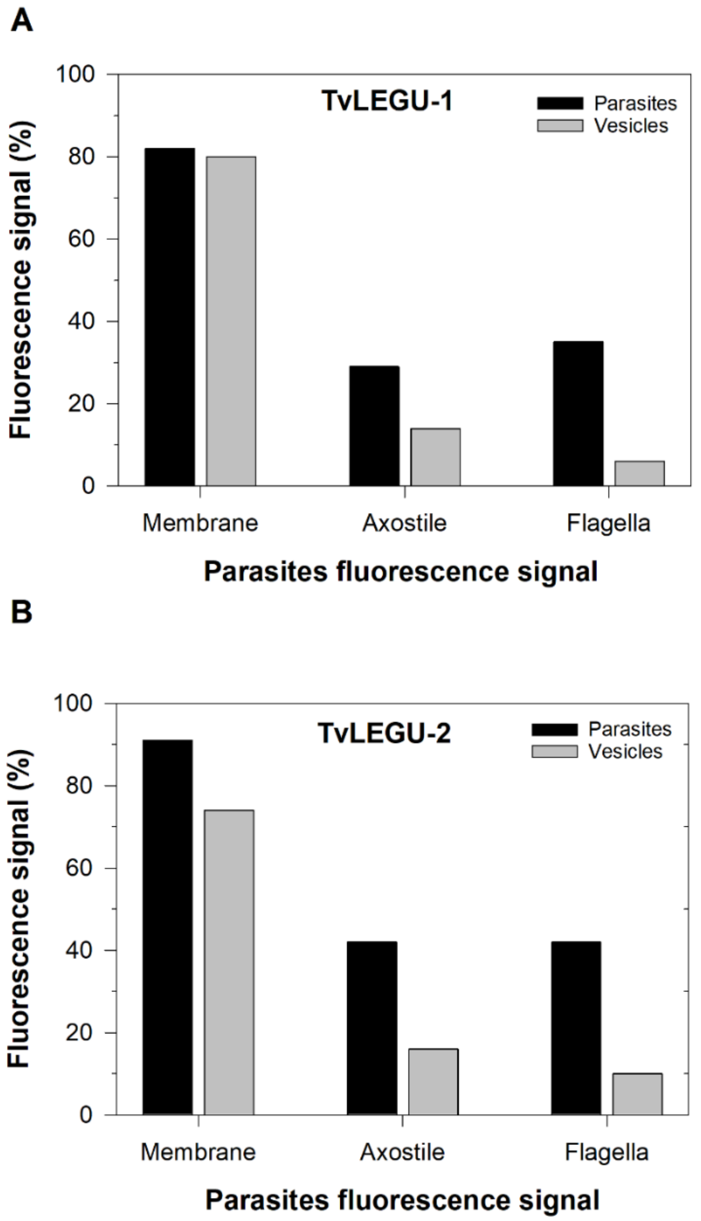

Supplementary Figure S6. The percentage distribution of parasite fluorescence signals for TvLEGU-1 and TvLEGU-2 in different parasite regions during secretion, as shown in Figures 12-15. Quantitative analysis of three ultra-resolution images, consisting of 20 to 26 optical sections obtained with the Zeiss LSM900 confocal microscope, was used to count all parasites (34 for TvLEGU-1 and 33 for TvLEGU-2) after 30 min secretion under HG conditions. The presence of TvLEGU-1 and TvLEGU-2 labels on the membrane, axostile, flagella, and vesicles, within the same parasite regions, was quantified by analyzing all optical sections for each label. This data is summarized in Supplementary Tables S3 and S4, respectively. Graphs were performed using Sigma-Plot 14.5.

Supplementary Table S1. Parasites exhibiting a TvLEGU-1 fluorescence signal during secretion assays conducted under various glucose conditions.

GR condition		Time (min)		
**Experiment**	**Image**	**0**	**30**	**60**
**I**	**IA**	14	18	18
	**IB**	14	19	12
**II**	**IIA**	12	19	14
	**IIB**	21	22	9
	**Total parasites[a]**	**61**	**78**	**53**

HG condition		Time (min)		
**Experiment**	**Image**	**0**	**30**	**60**
**I**	**IA**	12	22	17
	**IB**	6	26	13
**II**	**IIA**	14	18	23
	**IIB**	13	14	29
	**Total parasites[a]**	**45**	**56**	**82**

[a]Total parasites used to estimate the TvLEGU-1 fluorescence intensity (Supplementary Figure S3) at 0, 30, and 60 min during secretion assays under various glucose conditions (GR and HG). Parasites were selected from two images (A, B) of two independent secretion assays experiments (I, II) either from glucose restriction (GR) or high glucose (HG) conditions.

Supplementary Table S2. Parasites exhibiting a TvLEGU-2 fluorescence signal during secretions assays conducted under various glucose conditions.

GR condition		**Time (min)**		
**Experiment**	**Image**	**0**	**30**	**60**
**I**	**IA**	11	22	17
	**IB**	16	26	13
**II**	**IIA**	15	18	23
	**IIB**	12	14	29
	**Total parasites[a]**	**54**	**80**	**82**

HG condition		**Time (min)**		
**Experiment**	**Image**	**0**	**30**	**60**
**I**	**IA**	10	7	23
	**IB**	15	9	26
**II**	**IIA**	21	11	25
	**IIB**	17	7	23
	**Total parasites[a]**	**63**	**34**	**97**

[a]Total parasites used to estimate the TvLEGU-2 fluorescence intensity (Supplementary Figure S3) at 0, 30, and 60 min during secretion assays under various glucose conditions (GR and HG). Parasites were selected from two images (A, B) of two independent secretion assays experiments (I, II) either from glucose restriction (GR) or high glucose (HG) conditions.

Supplementary Table S3. Fluorescence signal (FS) of TvLEGU-1 in parasites and vesicles at 30 min during the secretion assays under high glucose conditions[a].

			**Parasites**				**Vesicles**				
				**Localization (w/FS)**					**Localization (w/FS)**		
	**Image**	**Optical Sections**	**Total**	**Membrane**	**Axostile**	**Flagella**	**Total**	**w/FS**	**Membrane**	**Axostile**	**Flagella**
	**A**	26	13	13	7	9	47	46	37	9	0
	**B**	20	9	7	1	1	67	11	8	0	3
	**C**	20	12	8	2	2	150	14	12	1	1
**Total**	3	66	34	28	10	12	264	71	57	10	4
**Percentage**			**100**	**82**	**29**	**35**		**100**	**80**	**14**	**6**

[a]To determine the fluorescence signal (FS) of TvLEGU-1 in parasites and their cellular location, three confocal microscopy images were analyzed (A from Figure 12, B and C from Figure 14). Total parasites identified in all the optical sections analyzed were taken as 100% to estimate the percentage distribution of the FS on membrane, axostile, or flagella. Protruding vesicles of parasites were identified, and those with FS were taken as 100% to estimate the FS of labeled vesicles located on the membrane, axostile, or flagella.

Supplementary Table S4. Fluorescence signal (FS) of TvLEGU-2 in parasites and vesicles at 30 min during the secretion assays under high glucose conditions[a].

			Parasites				Vesicles				
				Localization (w/FS)					Localization (w/FS)		
	**Image**	**Optical Sections**	**Total**	**Membrane**	**Axostile**	**Flagella**	**Total**	**w/FS**	**Membrane**	**Axostile**	**Flagella**
	**B**	20	9	8	3	3	67	30	23	3	4
	**C**	20	12	10	3	5	150	42	33	5	4
	**D**	25	12	12	8	6	62	45	30	11	4
**Total**	3	65	33	30	14	14	279	117	86	19	12
**Percentage**			**100**	**91**	**42**	**42**		**100**	**74**	**16**	**10**

[a]To determine the fluorescence signal (FS) of TvLEGU-2 in parasites and their cellular location, three confocal microscopy images were analyzed (B and C from Figure 14 and D from Figure 13). Total parasites identified in all the optical sections analyzed were taken as 100% to estimate the percentage distribution of the FS on membrane, axostile, or flagella. Protruding vesicles of parasites were identified, and those with FS were taken as 100% to estimate the FS of labeled vesicles located on the membrane, axostile, or flagella.
